# Inorganic, Synthetic, Natural, and Innovative Hybrid Hydrogen Sulfide Donors and Inhibitors of Its Biosynthesis in the Treatment of Central and Peripheral Nervous System Injuries: A Systematic Analytical Review

**DOI:** 10.3390/ijms262411842

**Published:** 2025-12-08

**Authors:** Stanislav Rodkin, Sergey Golovin, Stanislav Bachurin, Anton Lisovin, Inna Vasilieva, Anastasia Tolmacheva, Vasilii Chulkov, Mitkhat Gasanov

**Affiliations:** 1 Research Laboratory “Medical Digital Images Based on the Basic Model”, Department of Bioengineering, Institute of Living Systems, Don State Technical University, 344000 Rostov-on-Don, Russia; 2 N.V. Sklifosovsky Institute of Clinical Medicine, Department of Polyclinic Therapy, I.M. Sechenov First Moscow State Medical University, 119435 Moscow, Russia; 3 Department of Faculty Therapy Named After Professor G.D. Zalessky, Novosibirsk State Medical University, Krasny Prospekt, 52, Department of Medical Rehabilitation, Novosibirsk Regional Clinical Hospital of War Veterans No. 3, Demyan the Poor, 71, 630005 Novosibirsk, Russia; tolmacheva_nastena@mail.ru; 4 Department of Hospital Therapy, Yaroslav-the-Wise Novgorod State University, Derzhavina St. 6, 173020 Veliky Novgorod, Russia

**Keywords:** hydrogen sulfide, hydrogen sulfide donors, traumatic brain injury, spinal cord injury, peripheral nervous system injury, neuron, glial cells, neuroprotection, H_2_S-releasing hybrids, blood–brain barrier, oxidative stress, neuroinflammation, cerebral ischemia, axonal regeneration, microglia polarization, brain edema, mitochondria-targeted donors, slow-release donors, neuropathic pain

## Abstract

Hydrogen sulfide (H_2_S) is a gasotransmitter that plays a crucial role in regulating pathological processes following injury to the central and peripheral nervous systems. This review systematizes current data on various classes of H_2_S donors and inhibitors of its biosynthesis in neurotrauma and related experimental models. Inorganic donors (e.g., NaHS, Na_2_S, and STS) rapidly suppress oxidative stress and inflammation, supporting the recovery of synaptic plasticity and cognitive function. Organic donors (e.g., GYY4137, ACS67, ACS84, SPRC, ADT-OH and its derivatives, S-memantine, and MTC) provide sustained H_2_S release, stabilize the blood–brain barrier, and exhibit antiapoptotic activity. Natural donors (e.g., DADS, DATS, and SAMe) demonstrate high biocompatibility, inhibit pyroptosis, and enhance antioxidant defense mechanisms. Hybrid systems—including nanoparticles and hydrogels—enable targeted delivery and prolonged action, thereby stimulating regeneration and angiogenesis. Thiol-activated donors (e.g., COS/H_2_S and AlaCOS) allow controlled H_2_S release, offering broad opportunities for precise modulation of its concentration within target tissues. Inhibitors (e.g., AOAA, PAG, oxamic hydrazide 1, L-aspartic acid, benserazide, and NSC4056) of H_2_S biosynthesis underscore the physiological importance of this gasotransmitter, as their administration enhances neuroinflammation and diminishes neuroprotection. The analysis reveals a general pattern: all classes of H_2_S donors effectively modulate key pathological mechanisms, differing in their rate, duration, and specificity of action. These findings highlight the therapeutic promise of H_2_S-based pharmacological agents in clinical neurotraumatology, while emphasizing the need for further research to optimize delivery systems, enhance efficacy, and minimize adverse effects.

## 1. Introduction

Neurotrauma, including traumatic brain injury (TBI) and spinal cord injury (SCI), represents one of the leading causes of disability and mortality worldwide, posing a significant threat to global healthcare systems [1,2]. In addition, injuries to the peripheral nervous system can also lead to severe and long-term health complications [3]. These conditions are characterized by the activation of a complex signaling network that induces oxidative stress, neuroinflammation, reactive gliosis, disruption of the structural and functional integrity of the blood–brain barrier (BBB), and degenerative alterations in neurons and surrounding glial cells, ultimately resulting in cell death [4].

Despite considerable progress in neurotraumatology, including the development of applied treatment strategies and advances in understanding molecular mechanisms, current therapeutic approaches remain limited. They are largely focused on symptomatic management and rehabilitation, while effective interventions aimed at suppressing secondary injury and stimulating neural tissue regeneration are lacking. This gap arises from the absence of a unified molecular–cellular concept of neurotrauma pathogenesis and the scarcity of clinically effective neuroprotective agents [5].

In this context, the study of endogenous gaseous signaling molecules such as hydrogen sulfide (H_2_S) has become particularly relevant. Extensive preclinical evidence demonstrates the fundamental role of H_2_S in modulating key signaling mechanisms that mediate neuroprotection through activation of antioxidant systems, stabilization of the BBB, attenuation of neuroinflammation, and suppression of cell death [6,7,8]. As a potent reducing agent, H_2_S directly scavenges reactive oxygen species (ROS) and inhibits free-radical reactions, thereby stabilizing cell membranes and maintaining neuronal energy homeostasis. H_2_S reaches its highest concentrations in the brain due to the activity of key enzymes—cystathionine β-synthase (CBS) and cystathionine γ-lyase (CSE)—which catalyze its synthesis. The enzyme 3-mercaptopyruvate sulfurtransferase (3-MST) also contributes to H_2_S production, although its role in maintaining H_2_S levels in nervous tissue is less pronounced [9]. Intracellular H_2_S homeostasis constitutes a dynamic regulatory system that responds sensitively to internal and external stimuli. Its biological actions are dose-dependent and predominantly neuroprotective, supporting neuronal survival and stabilizing cellular processes. However, excessive H_2_S production can have deleterious consequences, contributing to mechanisms of secondary injury [10,11,12].

Thus, H_2_S-associated agents hold significant potential for integration into modern therapeutic strategies for neurotrauma, offering prospects for effective stabilization and regeneration of damaged neurons and glial cells. Nevertheless, despite an extensive “scientific library” of experimental data, translation into clinical practice remains challenging. The absence of a unified concept of H_2_S-dependent neuroprotection—caused by the heterogeneity of experimental models, research protocols, doses, and modulators—has led to a fragmented understanding of this gasotransmitter’s role under conditions of neuronal stress [13]. To date, most studies have focused on isolated effects of individual H_2_S donors or inhibitors, without comprehensive analysis of their structural–functional features or interactions within the intricate network of signaling pathways activated during neurotrauma. Moreover, unresolved questions regarding biocompatibility, targeted delivery, and minimization of adverse effects continue to fuel scientific debate over the most effective and safe H_2_S-based neuroprotective agent, thereby delaying the clinical translation of preclinical findings [14,15,16].

In our previous review studies, we systematically analyzed the role of gasotransmitters, including H_2_S, in the pathogenesis of various neurological disorders such as neurodegenerative diseases, as well as injuries to the central and peripheral nervous systems [10,17,18]. Furthermore, we have accumulated substantial experimental experience investigating the effects of several H_2_S donors on the regulation of cell death mechanisms following TBI [19,20,21,22]. These data provided the foundation for the present review, which is based on a comprehensive analysis and conceptual synthesis of existing information on H_2_S donors and inhibitors.

The novelty of this review lies in the systematic qualitative analysis of various classes of H_2_S modulators—ranging from inorganic and organic donors to hybrid nanosystems and biosynthesis inhibitors—with a particular emphasis on identifying common patterns in their mechanisms of action, including protein persulfidation, activation of antioxidant systems, receptor interactions, modulation of neuroinflammation, and regenerative processes. This work aims to compile and systematize the biological effects of H_2_S-associated compounds in diverse experimental models of nervous tissue injury, including TBI, SCI, peripheral nerve damage, stroke, ischemia/reperfusion (I/R), and neurodegenerative diseases. Such an integrative approach enables the identification of shared molecular and cellular mechanisms of H_2_S action depending on the type of donor and supports the development of a unified conceptual model of H_2_S-dependent signaling in neurotrauma, thereby facilitating the selection of optimal delivery strategies.

The objective of this review is to conduct a comprehensive analysis of key patterns in the biological effects of H_2_S modulators under neurotraumatic conditions, with a focus on their structural characteristics, mechanisms of action, and therapeutic potential. We will examine inorganic, organic, natural, and hybrid H_2_S donors, as well as inhibitors of its biosynthesis, emphasizing differences in release kinetics, duration of action, cellular and molecular effects, biocompatibility, and targeted activity toward injured neural tissue.

## 2. Methods

This systematic review was conducted in strict accordance with the PRISMA-ScR guidelines [23], ensuring a structured and transparent analytical approach. A comprehensive literature search was performed using major international databases, including PubMed, Scopus, Web of Science, and Google Scholar. The scoping review includes 192 scientific sources, ≈70% of which were published between 2022 and 2025. Notably, approximately ≈70% of the studies cited in the “Results” section were published during this same period, highlighting both the high relevance and rapid expansion of research on the role of H_2_S in neuroprotection following traumatic injuries to the central and peripheral nervous systems. This further underscores the need for systematic conceptualization of these data, which was first accomplished in the present work.

Although the literature search prioritized recent publications (2022–2025), studies published from 2011 onward were also included to ensure comprehensive coverage and adequate contextual depth. This approach enabled the integration of key early investigations with the latest findings, providing a balanced representation of the evolution of scientific knowledge regarding H_2_S-dependent neuroprotective mechanisms. Such a strategy is essential for developing a unified molecular–cellular framework that explains the roles of various H_2_S donors and inhibitors of its biosynthetic enzymes in the pathogenesis of neurotrauma.

### 2.1. Search Strategy and Selection of Publications

The literature search employed keywords directly related to the topic of this review: “hydrogen sulfide”, “hydrogen sulfide donors”, “neurotrauma”, “traumatic brain injury”, “spinal cord injury”, “peripheral nerve injury”, “neuroprotection”, and “H_2_S-associated compounds”. These terms were combined using Boolean operators (AND, OR, NOT) to construct targeted queries that maximized relevance to the research objectives. Typical search strings included combinations such as “hydrogen sulfide AND neurotrauma” and “H_2_S donors AND spinal cord injury” (Supplementary Materials Table S1), enabling the identification of studies addressing both the fundamental and applied aspects of H_2_S in neurotraumatology.

The selection process was conducted in several stages. Initially, titles and abstracts were screened for relevance, followed by full-text analysis of eligible studies. Each publication was evaluated for methodological rigor, scientific validity, relevance, and consistency of results with the objectives of this review.

Inclusion criteria:

1. Studies reporting the biological effects of H_2_S donors in neurotrauma models or related experimental paradigms.
2. Publications providing mechanistic or interdisciplinary insights linking H_2_S to molecular pathways of neuroprotection.
3. Original research articles or review papers aligned with the thematic scope of this study.

Exclusion criteria:

1. Publications with significant methodological limitations (e.g., absence of control groups, inadequate statistical analysis, or poor reproducibility).
2. Non-peer-reviewed materials.
3. Insufficient informativeness or weak relevance to the subject of the review.

**Figure 1:**
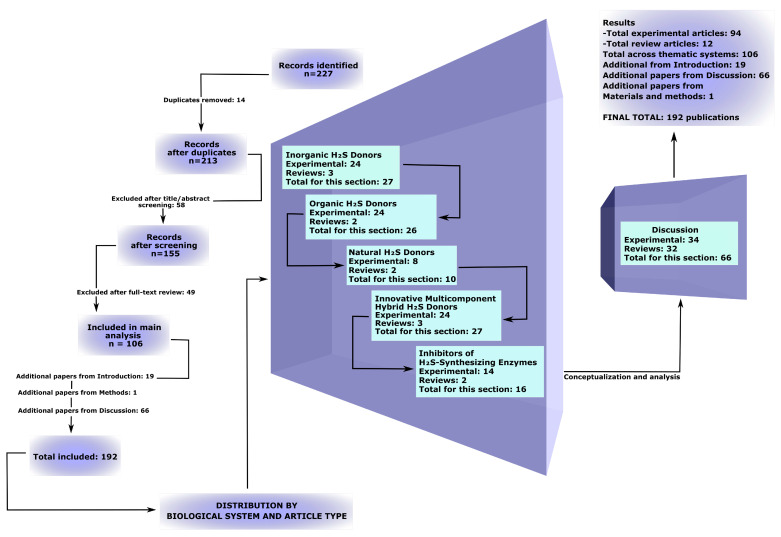
Expanded PRISMA-ScR flowchart illustrating the process of study identification, screening, eligibility assessment, and inclusion in the review, with details by publication type in the relevant sections.

The database search across PubMed, Web of Science, and Scopus initially identified 227 publications. After removing 14 duplicates, 213 records remained. During the title and abstract screening, 58 studies were excluded, followed by an additional 49 exclusions after full-text assessment. Ultimately, 106 scientific articles were included in the main qualitative analysis. In addition, 19 studies were included in the Introduction but were not used in the subsequent main results sections, and 1 source was included in the Materials and Methods (PRISMA-ScR guideline). Furthermore, 66 additional publications were incorporated into the Discussion to support interpretation of the qualitative analysis (Supplementary Materials Table S2). The detailed selection process is presented in the extended PRISMA-ScR flow diagram (Figure 1).

### 2.2. Quality Assessment and Decision-Making

Decisions on inclusion of studies were made by the authors independently based on a comprehensive assessment of their quality, relevance, and information content. Standardized quality assessment criteria were applied, including evaluation of methodological rigor, clarity of objectives and conclusions, and the reliability of presented data. This approach ensured consistency, transparency, and scientific validity throughout the analytical process.

## 3. Results

### 3.1. Inorganic H_2_S Donors

Inorganic H_2_S salts are the simplest and most widely used sources of exogenous H_2_S in experimental models of nervous system injury. They allow rapid simulation of H_2_S pharmacodynamics to study its biological effectors and molecular mechanisms of interaction with various extracellular and intracellular targets. The rapid release of H_2_S provides pronounced neuroprotection in ischemic stroke, TBI, and SCI by limiting the wave of secondary damage mediated through oxidative stress, apoptosis, and inflammation. However, the short-lived concentration peak that determines their transient action imposes serious limitations on their translation into clinical practice and stimulates the development of novel donors with controlled H_2_S release [24,25].

**Figure 2:**
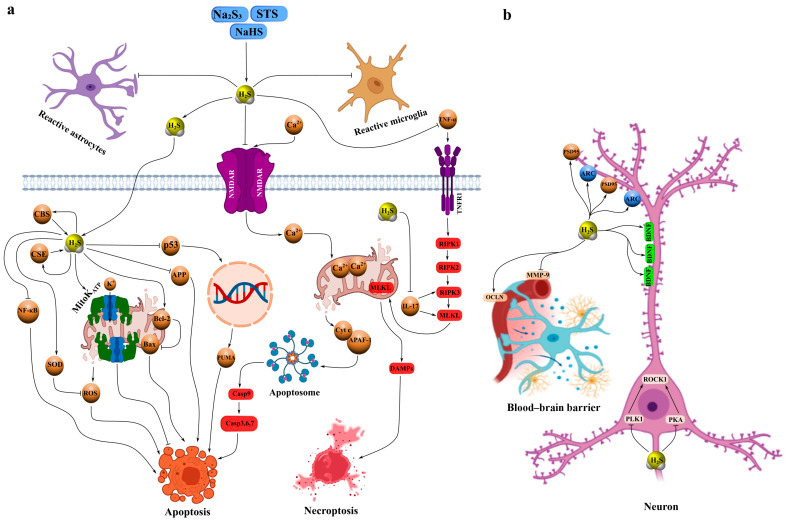
Schematic representation of the neuroprotective mechanisms of action of H_2_S donors based on fast-releasing donors NaHS, Na_2_S, and STS in models of neurotrauma, such as TBI, I/R, and peripheral nerve injuries. (**a**) Antiapoptotic, anti-inflammatory, and antioxidant H_2_S-dependent neuroprotective effects. The donors Na_2_S, STS, and NaHS rapidly release H_2_S, which inhibits NMDAR, reducing Ca^2+^ influx into neurons and suppressing the activation of reactive astrocytes and microglia. H_2_S activates H_2_S-synthesizing enzymes—CBS and CSE—enhancing endogenous H_2_S production. Additionally, H_2_S inhibits the transcription factors NF-κB and p53, the latter of which activates proapoptotic genes such as PUMA, suppresses the expression of APP, increases levels of the antiapoptotic protein Bcl-2, and activates MitoK_ATP_, contributing to antioxidant defense. H_2_S also inhibits pro-inflammatory cytokines IL-17 and TNF-α; TNF-α activates TNFR1, triggering a necroptosis cascade via RIPK1, RIPK2, RIPK3, and MLKL, leading to the release of DAMPs. Excessive Ca^2+^ induces the release of cytochrome c from mitochondria, its binding to APAF-1, and activation of the apoptosome, enhancing apoptosis. (**b**) H_2_S-dependent effects on synaptic plasticity and BBB stabilization. H_2_S upregulates the expression of synaptic proteins PSD-95, ARC, and BDNF, promoting improved dendritic branching, spine density, and restoration of cognitive functions. It inhibits the phosphorylation and activation of ROCK2 by suppressing PLK1 and PKA, reducing the expression of LDH, NSE, and intracellular Ca^2+^. Additionally, H_2_S stabilizes the BBB by inhibiting MMP-9 and enhancing the stability of OCLN, reducing brain edema, barrier permeability, and lesion volume in TBI and I/R models. Arrows with pointed ends indicate activation, while blunt ends indicate inhibition.

Sodium hydrosulfide (NaHS) is a classical fast-releasing donor of H_2_S that exhibits a broad spectrum of neuroprotective effects in various models of neurotrauma. For example, in TBI, administration of NaHS increased brain H_2_S levels, inhibited oxidative stress, and reduced the expression of injury biomarkers. These effects were accompanied by elevated levels of synaptic proteins, enhanced dendritic branching and spine density, and significant improvements in cognitive and motor performance, including reduced deficits in the Barnes maze and novel object recognition tests. The underlying mechanisms are believed to involve H_2_S-dependent modulation of N-methyl-D-aspartate (NMDA) receptor activity, glutamate and Ca^2+^ homeostasis, and regulation of microtubule-associated proteins and calcium/calmodulin-stimulated protein kinase II (CaMKII) (Figure 2a, Table 1) [26].

Similarly, NaHS injections increased endogenous H_2_S levels, suppressing neuroinflammation and oxidative stress induced by TBI while upregulating brain-derived neurotrophic factor (BDNF), activity-regulated cytoskeleton-associated protein (ARC), and postsynaptic density protein 95 (PSD-95)—proteins essential for synaptic plasticity and cognitive recovery (Figure 2b) [27]. In a controlled cortical impact model, NaHS demonstrated pronounced neuroprotective effects by reducing brain edema, BBB permeability, and lesion volume through activation of mitochondrial ATP-sensitive K^+^ channels (mitoK_ATP), enhancement of antioxidant defense, and inhibition of free radical generation (Figure 2a, Table 1) [28].

Intrathecal administration of NaHS prevented dopaminergic neuronal loss in the midbrain following chronic constriction injury of the sciatic nerve, likely via inhibition of IL-17-induced necroptosis and suppression of mixed lineage kinase domain-like protein (MLKL) expression, tyrosine hydroxylase activity, and neuronal firing frequency [29]. In peripheral nerve injury models, NaHS reduced glutamate levels in the spinal cord and cortical neuronal firing frequency by modulating astrocytic function and excitatory amino acid transporter 2 (EAAT2) expression [30]. Moreover, NaHS attenuated degeneration of dorsal root ganglion neurons and sciatic nerve axons in diabetic neuropathy by activating antioxidant enzymes such as superoxide dismutase (SOD) and superoxide dismutase 2 (SOD2) and downregulating aldose reductase expression (Figure 2a, Table 1) [31].

In cerebral I/R models, NaHS inhibited rho-associated protein kinase 2 (ROCK2) expression and activation through phosphorylation at Thr436 and Ser575. For instance, in bilateral carotid artery occlusion, NaHS markedly reduced lactate dehydrogenase (LDH), neuron-specific enolase (NSE), ROCK2, and bcl-2-associated x protein (Bax) expression, while increasing b-cell lymphoma 2 (Bcl-2) levels and suppressing oxidative stress, leading to improved cognitive outcomes. Notably, these effects were abolished by mutations at Thr436 or Ser575 [32]. In cultured hippocampal neurons, NaHS inhibited phosphorylation of ROCK2, polo-like kinase 1 (PLK1), and protein kinase A (PKA), decreased ROCK2, LDH, NSE, and intracellular Ca^2+^ levels, and enhanced cell viability under hypoxia/reoxygenation conditions via similar phosphorylation sites [33]. Furthermore, NaHS exhibited neuroprotective properties in cardiac arrest models by reducing brain edema and BBB disruption through inhibition of matrix metalloproteinase-9 (MMP-9) expression and stabilization of occludin (Figure 2b, Table 1) [34].

In lateral percussion models of TBI, subchronic NaHS administration prevented hypertension, vascular dysfunction, and oxidative stress in the aorta while restoring expression of H_2_S-synthesizing enzymes and endothelial nitric oxide synthase (eNOS) phosphorylation. These effects were abolished by co-administration of L-NAME, confirming the NO-dependence of the mechanism [35]. In a related study, NaHS prevented tachycardia, hypertension, and sympathetic hyperactivity, and reduced vasopressor responses to norepinephrine [36]. Additionally, after severe TBI, NaHS restored the expression of key H_2_S-synthesizing enzymes, such as CBS and CSE, in neural tissue, correlating with posttraumatic stage and brain region [37]. Microinjections of NaHS into the hypothalamus were also shown to lower arterial pressure and sympathetic activity by restoring n-methyl-d-aspartate receptor subunit 1 (NMDAR1) disulfide bonds in ischemic stroke models [38]. Moreover, NaHS reduced demyelination, apoptosis, and expression of platelet-derived growth factor receptor alpha (PDGFRα), glial fibrillary acidic protein (GFAP), nuclear factor kappa b (NF-κB), and interleukin-1 beta (IL-1β), while improving locomotor and cognitive functions (Figure 2a, Table 1) [39].

Another fast-releasing H_2_S donor, sodium sulfide (Na_2_S), has also demonstrated promising neuroprotective potential. In models of intracerebral hemorrhage, Na_2_S inhibited neurotoxic processes such as neuronal death, axonal degradation, impaired axonal transport, and inflammation mediated by CXC motif ligand 2. Interestingly, another sulfide donor, Na_2_S_3_, did not prevent neuronal death or inflammatory activation but otherwise produced effects similar to Na_2_S [40]. In global cerebral I/R, Na_2_S inhibited caspase-3 activity through persulfidation of Cys163, reducing mortality and mitigating neurological deficits. This neuroprotective effect appeared to be mediated by sodium thiosulfate (STS), as Na_2_S administration significantly increased plasma and brain thiosulfate levels, transported across membranes by the sodium sulfate cotransporter SLC13A4 (NaS-2) (Table 1) [41].

Our previous studies also demonstrated that Na_2_S reduced the number of TUNEL-positive neurons and glial cells after TBI and axotomy, likely through H_2_S-dependent downregulation of the proapoptotic protein p53. Conversely, inhibition of CBS with aminooxyacetic acid (AOAA) enhanced apoptosis, confirming CBS as a key mediator of H_2_S-induced neuroprotection [19]. In subsequent work, we found that p53 colocalized with damaged neurons and glial cells exhibiting nuclear fragmentation after severe TBI [21]. Moreover, Na_2_S decreased the expression of inducible nitric oxide synthase (iNOS) and amyloid precursor protein (APP) in injured neural tissue at 24 h and 7 days post-TBI, whereas AOAA increased their expression. Computational modeling further revealed specific binding sites for H_2_S and its derivatives on iNOS and APP, supporting molecular mechanisms underlying H_2_S-mediated neuroprotection (Figure 2a, Table 1) [20].

Sodium thiosulfate (STS) is also considered a promising inorganic H_2_S donor. In glial cells, STS increased H_2_S and glutathione (GSH) levels while decreasing tumor necrosis factor-alpha (TNF-α), interleukin-6 (IL-6), and activation of the p38 mitogen-activated protein kinase (p38 MAPK) and nuclear factor κB (NF-κB) pathways, thus exerting time- and dose-dependent neuroprotective effects [42]. However, other studies reported no significant effects of STS on brain tissue damage in hemorrhagic shock models, likely due to an intact BBB preventing its penetration [43]. STS has also been shown to stimulate vascular endothelial growth factor (VEGF)-dependent angiogenesis in hindlimb ischemia by inhibiting mitochondrial respiration and promoting glycolysis in endothelial cells (Table 1) [44].

In our experiments, STS reduced neuronal and astrocytic apoptosis after severe TBI through inhibition of p53-dependent cell death pathways. Conversely, inhibition of H_2_S synthesis enhanced p53 expression and apoptosis, confirming the modulatory role of H_2_S. Molecular dynamics simulations indicated that H_2_S does not form stable complexes with p53 but induces wave-like fluctuations in its conformational dynamics, particularly through weak van der Waals interactions with residues such as Arg248. This may reduce p53’s DNA-binding affinity and modulate its proapoptotic activity, thereby conferring neuroprotection under ischemic conditions associated with low pH, typical of the TBI microenvironment. Such pH-dependent effects of H_2_S, manifested as increased neutral H_2_S species during acidosis, may act as a metabolic sensor, indirectly regulating p53 transcriptional activity and promoting neuronal survival after injury (Table 1) [21].

Recent interest has also focused on thiol-activated carbonyl sulfide/hydrogen sulfide (COS/H_2_S) donors with fluorescent properties, enabling real-time tracking of H_2_S generation in vivo [45]. These COS/H_2_S donors can additionally serve as molecular platforms for the targeted delivery of fluorophores or therapeutic agents [46]. Among these, alanyl-carbonyl sulfide (AlaCOS) represents a particularly promising donor capable of tissue-specific H_2_S release upon activation by aminopeptidase N (APN). Incorporation of a coumarin fluorophore into AlaCOS permits visualization of H_2_S generation, offering a valuable tool for both mechanistic and translational neurotrauma research (Table 1) [47].

**Table 1:** Neuroprotective effects of inorganic H_2_S donors in experimental models of nervous system injury. Arrows ↑ and ↓ denote increase and decrease, respectively. Abbreviations. Main active substance: H_2_S—hydrogen sulfide. Inorganic H_2_S donors: NaHS—sodium hydrosulfide; Na_2_S—sodium sulfide; Na_2_S_3_—sodium trisulfide; STS—sodium thiosulfate; COS/H_2_S—thiol-activated H_2_S donor based on carbonyl sulfide; AlaCOS—alanine-containing thiol-activated H_2_S donor. Main injury models: TBI—traumatic brain injury; BBB—blood–brain barrier; ICH—intracerebral hemorrhage; I/R—ischemia/reperfusion; CPR—cardiopulmonary resuscitation; OGD/R—oxygen-glucose deprivation/reoxygenation; CCI—chronic constriction injury of the sciatic nerve; CPZ—cuprizone (demyelination model); HLI—hindlimb ischemia; MCAO—middle cerebral artery occlusion. Key molecular targets and signaling pathways: K_ATP channels—ATP-sensitive potassium channels of mitochondria; ROCK2—Rho-associated kinase 2; PLK1—polo-like kinase 1; PKA—protein kinase A; p53—tumor suppressor p53; p-eNOS—phosphorylated endothelial NO synthase; NR1/NMDAR1—subunit 1 of NMDA receptor; CaMKII—calcium/calmodulin-dependent protein kinase II; NF-κB—nuclear factor kappa B; p38 MAPK—p38 mitogen-activated protein kinase. Enzymes of H_2_S synthesis and inhibitors: CBS—cystathionine-β-synthase; CSE—cystathionine-γ-lyase; AOAA—aminooxyacetic acid; L-NAME—Nω-nitro-L-arginine methyl ester. Protein markers of damage and inflammation: Bax—proapoptotic Bcl-2 family protein; Bcl-2—antiapoptotic Bcl-2 family protein; LDH—lactate dehydrogenase; NSE—neuron-specific enolase; iNOS—inducible NO synthase; APP—amyloid precursor protein; GFAP—glial fibrillary acidic protein; PDGFRα—platelet-derived growth factor receptor α; MLKL—mixed lineage kinase domain-like protein. Cytokines and inflammatory mediators: IL-1β—interleukin-1β; IL-6—interleukin-6; IL-17—interleukin-17; TNFα—tumor necrosis factor α. Neurotrophic and synaptic proteins: BDNF—brain-derived neurotrophic factor; ARC—activity-regulated cytoskeleton-associated protein; PSD-95—postsynaptic density protein 95. Transporters and barrier proteins: EAAT2—excitatory amino acid transporter 2; occludin—tight junction protein; MMP-9—matrix metalloproteinase-9; SLC13A4/NaS-2—thiosulfate transporter. Antioxidant systems: GSH—reduced glutathione; SOD/SOD2—superoxide dismutase 1 and 2. Apoptosis markers: TUNEL—terminal deoxynucleotidyl transferase dUTP nick end labeling. Cell lines and experimental systems: SH-SY5Y—human neuroblastoma cell line; THP-1—human monocyte cell line; U373—human astrocyte cell line; HUVEC—human umbilical vein endothelial cells; HeLa—human cervical carcinoma cell line; RAW264.7—mouse macrophage cell line. Other: PVN—paraventricular nucleus of the hypothalamus; PSH—post-stroke sympathetic hyperactivity; BP—blood pressure; LPS—lipopolysaccharide; IFNγ—interferon gamma; VEGF—vascular endothelial growth factor; VEGFR2—VEGF receptor 2; PFKFB3—6-phosphofructo-2-kinase/fructose-2,6-bisphosphatase 3; eNOS—endothelial NO synthase; EdU—5-ethynyl-2′-deoxyuridine (proliferation marker), ERG—endothelial regulatory gene; 3PO—PFKFB3 inhibitor; 2-DG—2-deoxyglucose; APN—aminopeptidase N; CA—carbonic anhydrase; CAM—chick embryo chorioallantoic membrane; NIR—near-infrared; MTT—3-(4,5-dimethylthiazol-2-yl)-2,5-diphenyltetrazolium bromide.

Donor	Model, Animals, Concentration/Dose	Main Effects	Reference
NaHS	TBI, mice, 3.1 mg·kg^−1^ for seven days	↑ H_2_S in brain, ↓ oxidative stress, ↑ synaptic proteins, improvement of dendrites and spines, restoration of cognitive and motor functions (Barnes maze, object recognition), modulation of NMDA, glutamate, Ca^2+^, CaMKII.	[26]
NaHS	TBI, mice, 50 µmol/kg body weight for 7 days	↓ neuroinflammation and oxidative stress, ↑ BDNF, ARC, PSD-95, restoration of cognitive functions.	[27]
NaHS	Controlled cortical impact, rats and mice, 3 mg/kg 5 min post-injury	↓ brain edema, BBB permeability, lesion volume; activation of mitochondrial K_ATP channels, antioxidant protection.	[28]
NaHS	Chronic constriction injury of sciatic nerve (CCI), 4.43 nmol/mouse, intrathecal	Prevention of dopaminergic neuron death via ↓ IL-17 → necroptosis, ↓ MLKL, tyrosine hydroxylase.	[29]
NaHS	Peripheral nerve injury, mice, 10 µmol/mL, i.p. daily for 14 days	↓ glutamate in spinal cord, ↓ impulsation in somatosensory cortex, astrocyte modulation, ↑ EAAT2.	[30]
NaHS	Diabetic neuropathy, rats, 50 µmol/kg/day i.p. for 2 weeks	↓ degeneration of ganglia and sciatic nerve axons, ↑ SOD/SOD2, ↓ aldose reductase.	[31]
NaHS	Cerebral I/R (bilateral carotid artery occlusion) in rats transfected with wild-type and mutant ROCK2 eukaryotic plasmids in hippocampus, 4.8 mg/kg	Inhibition of ROCK2 (phosphorylation Thr436/Ser575), ↓ LDH, NSE, Bax, ROCK2; ↑ Bcl-2, ↓ oxidative stress, improved cognitive functions.	[32]
NaHS	Hypoxia/reoxygenation (hippocampal neuron culture), 50, 100 and 200 µmol/L	↓ ROCK2 via PLK1 and PKA, ↓ Ca^2+^, LDH, NSE; ↑ cell viability.	[33]
NaHS	Cardiac arrest, rats, 0.5 mg/kg i.v. at start of CPR, then maintenance infusion (1.5 mg·kg^−1^·h^−1^) for 6 h after ROSC	↓ brain edema, BBB degradation, ↓ MMP-9, stabilization of occludin.	[34]
NaHS	Lateral fluid percussion TBI (subchronic), rats, 3.1 mg/kg i.p. daily for six days	Prevention of post-traumatic hypertension, vascular dysfunction, aortic oxidative stress; restoration of H_2_S-synthesizing enzymes and p-eNOS (blocked by L-NAME).	[35]
NaHS	Lateral fluid percussion TBI, rats, 3.1 and 5.6 mg/kg i.p. for seven days	↓ tachycardia, hypertension, sympathetic hyperactivity, restoration of vasopressor responses to noradrenaline.	[36]
NaHS	TBI, lateral fluid percussion, rats, 3.1 mg/kg i.p. for seven days	Restoration of CBS and CSE levels in nervous tissue.	[37]
NaHS	Patients with ischemic stroke (MCI vs. NMCI)—no NaHS administered (only endogenous H_2_S and noradrenaline measured in plasma); rats: focal ischemic stroke (MCAO 90–120 min), bilateral microinjections of NaHS µM/100 nl into PVN	Patients: malignant infarction and PSH → ↓ endogenous H_2_S, ↑ plasma noradrenaline, positive correlation with damage markers, ↓ one-year survival. Rats: NaHS completely abolished PSH, ↓ BP and renal sympathetic activity, eliminated AOAA effect, restored disulfide bonds of NR1 NMDA receptor.	[38]
NaHS	Experimental multiple sclerosis model in rats induced by cuprizone (CPZ), rats, 50 and 100 µmol/kg, 14 days	↓ demyelination, apoptosis, PDGFRα, GFAP, NF-κB, IL-1β; improved locomotion and cognitive functions, but at 100 µmol/kg, the effect of NaHS decreased.	[39]
Na_2_S, Na_2_S_3_	Intracerebral hemorrhage (intrastriatal collagenase injection), mice, 25 µmol/kg i.p. 30 min before ICH induction	Na_2_S: significant improvement of sensorimotor functions, ↓ striatal neuron death, protection of axons and axonal transport, ↓ inflammatory mediators. Na_2_S_3_: protection of axons and axonal transport (comparable to Na_2_S), but no protection of neurons or suppression of inflammation; no improvement of sensorimotor functions.	[40]
Na_2_S, STS	1. In vitro: OGD/R in SH-SY5Y and primary mouse cortical neurons, Na_2_S 0.5 mmol/L (500 µM) added 5 h after OGD, STS 0.25 mmol/L (250 µM); 2. In vivo: global cerebral ischemia–reperfusion, mice, STS 10 mg/kg i.v. 1 min after reperfusion start + 10 mg/kg/day for 7 days	Na_2_S: almost no protection by itself; entire neuroprotective effect fully mediated by rapid oxidative metabolite—thiosulfate (increased thiosulfate, not H_2_S, in plasma and brain). STS: fully reproduces and substitutes all protective effects of Na_2_S; significantly ↑ cell and animal survival, improved neurological functions; antiapoptotic action via persulfidation of Cys163 of caspase-3; cellular uptake via NaS-2 (SLC13A4).	[41]
Na_2_S	1. TBI (controlled cortical impact), mice, 0.1 mg/kg daily i.p. for 7 days; 2. Axotomy (complete axon transection) in mechanoreceptor neuron, freshwater crayfish Astacus leptodactylus, 250 µM	↓ expression and nuclear translocation of p53 in neurons and glial cells at 24 h and 7 days post-TBI; ↓ apoptosis (TUNEL+), ↓ Bax, protection of neurons and glial cells; in axotomy model—↓ nuclear p53 in cytoplasm, axon and dendrites of motoneurons, ↓ apoptosis of satellite glia. Opposite effect with AOAA.	[19]
Na_2_S	1. TBI (controlled cortical impact), mice, 0.1 mg/kg daily i.p. for 7 days; 2. Axotomy, freshwater crayfish Astacus leptodactylus, 250 µM post-axotomy, assessed at 8 h	Significant ↓ iNOS and APP expression in neurons and astrocytes, ↓ apoptosis; in axotomy— ↓ iNOS and APP in motoneurons and axons. Opposite effect with AOAA.	[20]
Na_2_S/STS	TBI (controlled cortical impact), mice, 1000 mg/kg post-injury, in silico	↓ apoptosis of neurons and astrocytes via p53 modulation (van der Waals interactions with Arg248), pH-dependent H_2_S effect as metabolic sensor.	[21]
NaHS, STS	In vitro: LPS/IFNγ-activated microglia and human THP-1 monocytes; IFNγ-activated astrocytes and U373 cells Concentration: 1–500 µM (optimal 100 µM, 8–12 h)	↑ H_2_S and GSH in glial cells, ↓ TNFα and IL-6 release, ↓ p38 MAPK and NF-κB activation, significant neuroprotection of differentiated SH-SY5Y (MTT and LDH).	[42]
STS	Hemorrhagic shock (30% blood volume withdrawal + 3 h hypotension) followed by resuscitation, pigs with atherosclerosis Dose: 0.1 g·kg^−1^·h^−1^ i.v. for first 24 h of resuscitation	No effect (intact BBB prevents penetration), no differences in CSE, CBS, oxytocin/receptor, GFAP, nitrotyrosine in PVN; only minimal perivascular edema.	[43]
STS	Hindlimb ischemia (HLI) in C57BL/6 and hypercholesterolemic LDLR^−^/^−^ mice, oral 2–4 g/L in drinking water (≈0.5–1 g/kg/day); in ovo angiogenesis model—chick embryo CAM, 500 µM; in vitro (HUVEC)	In vivo: stimulation of VEGF-dependent angiogenesis via inhibition of mitochondrial respiration and stimulation of glycolysis, ↑ capillary density and endothelial proliferation (EdU+/ERG+), ↓ muscle injury area. Optimal dose 2 g/L (4 g/L less effective → narrow therapeutic window). Effect only in peripheral tissues. In vitro: ↑ proliferation and migration, ↑ H_2_S and protein persulfidation, ↓ mitochondrial respiration → ↑ glycolysis and ATP, ↑ PFKFB3, eNOS, VEGFR2. Effect completely abolished by 3PO and 2-DG.	[44]
COS/H_2_S	In vitro (buffer + CA), live HeLa cells, in vivo in rats (subcutaneous alginate gel)	Thiol-dependent H_2_S release (>60%) + fluorescence turn-on. Palette: blue, yellow, orange, red and NIR. Direct correlation of fluorescence and H_2_S (electrode). Visualization of H_2_S in live cells and subcutaneously in rats.	[45]
AlaCOS	In vitro, LPS-stimulated RAW264.7 macrophages in full-thickness skin wound model in mice	Tissue-specific H_2_S delivery, activation by aminopeptidase N (APN), built-in coumarin fluorophore for imaging.	[47]

### 3.2. Organic Synthetic H_2_S Donors

Organic synthetic H_2_S donors have been developed to provide slow, controlled, and more physiologically relevant H_2_S release, enabling them to mimic endogenous mechanisms of this gasotransmitter production. Owing to their modifiable chemical structures and diverse release mechanisms, these donors offer highly tunable pharmacokinetics with respect to onset, duration of action, and tissue specificity. In models of TBI, SCI, cerebral ischemia–reperfusion, neuropathic pain, and neurodegenerative diseases, they exhibit sustained and prolonged neuroprotection with comprehensive effects on cytotoxic processes. In addition, their high blood–brain barrier permeability and significantly lower toxicity compared to inorganic salts make this class of compounds the most promising candidates for further preclinical and clinical studies aimed at developing effective neuroprotective drugs [24,25,48,49].

GYY4137 is a slow-releasing H_2_S donor that has demonstrated neuroprotective effects in numerous pathological models of both the central and peripheral nervous systems, including neurotrauma. For example, in a spinal cord I/R model, administration of GYY4137 reduced neuronal loss through inhibition of cell death signaling mechanisms involving Bax, bcl-2-associated death promoter (Bad), caspase-mixed lineage kinase domain-like protein (caspase-MLKL), phosphorylated receptor-interacting protein 1/3 (p-RIP1/3), nucleotide-binding oligomerization domain-like receptor protein 3 (NLRP3), and pro-inflammatory factors. It also prevented Nissl body degradation, stabilized BBB permeability, and attenuated neuroinflammation [50]. The GYY4137-mediated protection of the BBB is thought to be related to H_2_S-dependent inhibition of autophagic degradation of occludin [51]. In the same I/R model, GYY4137 blocked apoptosis through suppression of p38 MAPK, extracellular signal-regulated kinase 1/2 (ERK1/2), and c-jun n-terminal kinase (JNK) phosphorylation (Table 2) [52].

**Figure 3:**
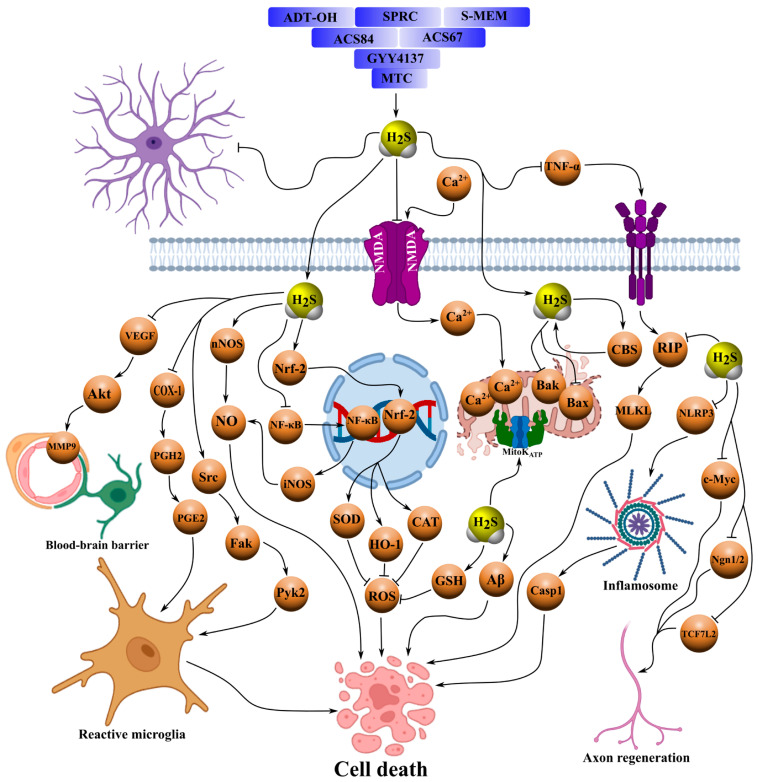
Schematic representation of the neuroprotective mechanisms of action of organic H_2_S donors, such as GYY4137, ACS67, ACS84, SPRC, ADT-OH and its derivatives, including ADT and ATB-346, S-memantine, and MTC, in various models of central and peripheral nervous system pathologies, including I/R, neurotrauma, Parkinson’s disease, Alzheimer’s disease, stroke, and TBI. These compounds serve as therapeutic agents for neuroprotection in various neurological disorders. They provide slow release of H_2_S, suppressing apoptosis by inhibiting signaling pathways involving Bax, Bad, caspases, MLKL, and p-RIP1/3, as well as phosphorylation of p38 MAPK, ERK1/2, and JNK, thereby reducing Ca^2+^-excitotoxicity. They mitigate oxidative stress by blocking nitrosative stress, lipid peroxidation, and MDA levels, and attenuate neuroinflammation by inhibiting NLRP3, pro-inflammatory factors (TNF-α, IL-1β, COX-2, NO, iNOS, and NOX4) and signaling pathways NF-κB, p38-, and JNK-MAPK. In parallel, H_2_S donors activate antioxidant systems, increasing levels of GSH, SOD, CAT, and GPx, and stimulating nuclear translocation of Nrf2. They modulate microglia, shifting them toward an anti-inflammatory phenotype through activation of AMPK, and maintain BBB integrity by stabilizing permeability, preventing degradation of occludin and tight junctions, and reducing edema and extravasation. Additionally, these compounds promote regeneration by enhancing axonal growth, neuronal and oligodendrocyte differentiation, vascular remodeling, and microglial migration through the Src/Fak/Pyk2 pathway. They inhibit sympathetic neurotransmission by suppressing [^3^H]-norepinephrine release via activation of K_ATP and prostanoid production and modulate endogenous H_2_S synthesis through activation of CBS. H_2_S donors improve cognitive functions by reducing astrogliosis, lipofuscin deposition, the Aβ_1–42_/Aβ_1–40_ ratio, and ultrastructural neuronal damage. Arrows with pointed ends indicate activation, while blunt ends indicate inhibition.

In isolated bovine ciliary body preparations, GYY4137 inhibited sympathetic neurotransmission by suppressing [^3^H]-norepinephrine release, partially through H_2_S generation, prostanoid production, and activation of K_ATP channels [53]. Moreover, GYY4137 reduced the number of TUNEL-positive retinal ganglion cells by inhibiting Ca^2+^-excitotoxicity induced by intravitreal NMDA injection [54]. It has also been reported that GYY4137 inhibits neuronal nitric oxide synthase (nNOS), thereby reducing nitrosative stress and neuronal death in Parkinson’s disease models (Figure 3, Table 2) [55].

Another notable H_2_S donor is ACS67, a latanoprost derivative with an H_2_S-releasing moiety. In a retinal ischemia model, intravitreal injection of ACS67 prevented pathological electroretinogram changes and attenuated the downregulation of retinal antigens and axonal proteins of the optic nerve, while enhancing the major antioxidant defense component—GSH [56]. Similarly, ACS67 inhibited electrically evoked [^3^H]-norepinephrine release in ocular tissues, an effect enhanced in the presence of the cyclooxygenase inhibitor flurbiprofen. This inhibitory effect was blocked by H_2_S synthesis inhibitors or K_ATP channel blockers (Figure 3, Table 2) [53].

A related H_2_S donor, ACS84—an L-DOPA derivative bearing an H_2_S-releasing group—also exhibits neuroprotective properties. For instance, ACS84 prevented neuronal death in the substantia nigra and inhibited lipid peroxidation, oxidative stress, and monoamine oxidase B (MAO-B) activity, thereby improving motor performance and surpassing the effects of NaHS and L-DOPA in Parkinson’s disease models [57]. ACS84 additionally promoted nuclear translocation of nuclear factor erythroid 2-related factor 2 (Nrf2), upregulated antioxidant defense enzymes, increased GSH levels, and reduced malondialdehyde (MDA) concentration [58]. In microglial cultures, ACS84 inhibited amyloid beta 1–40 (Aβ_1–40_)-induced cytotoxicity through H_2_S-dependent suppression of nitric oxide (NO), TNF-α, and p38- and JNK-MAPK signaling, thereby preventing mitochondrial dysfunction (Figure 3, Table 2) [59].

The sulfur-containing amino acid SPRC (ZYZ-802) modulates endogenous H_2_S synthesis via CBS activation. In Alzheimer’s disease models, SPRC reduced astrogliosis, lipofuscin accumulation, and the amyloid beta 1–42/amyloid beta 1–40 (Aβ_1–42_/Aβ_1–40_) ratio through H_2_S-associated inhibition of NF-κB and mitogen-activated protein kinase (MAPK) signaling, while increasing CBS activity and H_2_S concentration. SPRC also suppressed Aβ-induced astrocyte activation, reducing TNF-α and nitrite production; these effects were blocked by CBS small interfering RNA (siRNA) or AOAA, protecting neurons from Aβ cytotoxicity [60]. It has been reported that SPRC mitigated cognitive impairment and ultrastructural neuronal damage by inhibiting TNF-α, cyclooxygenase-2 (COX-2), ERK1/2, and NF-κB following Aβ_25–35_ injection [61]. In ischemic stroke models, SPRC upregulated cluster of differentiation 24 (CD24) expression via the CBS/H_2_S signaling pathway, suppressing NF-κB and enhancing src/focal adhesion kinase/proline-rich tyrosine kinase 2 (Src/Fak/Pyk2)-dependent microglial migration [62]. Furthermore, SPRC prevented hippocampal H_2_S depletion by inhibiting TNF-α, tumor necrosis factor receptor 1 (TNFR1), Aβ, and NF-κB during neuroinflammation, thereby improving cognitive performance (Figure 3, Table 2) [63].

ADT-OH and its derivative anethole trithione (ADT) are slow H_2_S-releasing compounds involved in regeneration and anti-inflammatory processes. Studies on cultured neural progenitor cells (NPCs) have shown that ADT-OH modulates differentiation into neurons and oligodendrocytes, suppresses astrogliogenesis and apoptosis, and promotes axonal growth via upregulation of beta-catenin (β-catenin), transcription factor 7-like 2 (TCF7L2), cellular myc (c-Myc), and neurogenin 1/2 (Ngn1/2) [64]. In spinal cord injury models, ADT enhanced regeneration by reducing glial scarring, neuronal loss, and microglial activation, while promoting vascular remodeling in the lesion site [65]. ADT-OH induced a shift in microglia toward an anti-inflammatory phenotype through adenosine monophosphate-activated protein kinase (AMPK) activation, suppressing M1 markers and enhancing M2 gene expression during neuroinflammation [66]. In a middle cerebral artery occlusion (MCAO) model, both ADT-OH and NaHS maintained BBB integrity, reduced infarct volume, edema, and Evans blue extravasation, and prevented tight junction degradation by inhibiting iNOS, IL-1β, MMP9, and nadph oxidase 4 (NOX4) via NF-κB-dependent mechanisms [67]. In a similar stroke model, ADT-OH reduced tissue plasminogen activator (tPA)-enhanced hemorrhage by inhibiting the protein kinase b/vascular endothelial growth factor/matrix metalloproteinase 9 (Akt/VEGF/MMP9) pathway (Table 2) [68].

ATB-346, an H_2_S-releasing naproxen derivative, attenuated brain edema, neuronal death, and inflammation while restoring neurotrophic factors in a controlled cortical impact model of TBI in mice, although direct inhibition of H_2_S synthesis was not evaluated (Figure 3, Table 2) [69].

S-memantine, a novel H_2_S donor derived from memantine with an ACS48 moiety, reduced cell death in neuroblastoma and cortical neurons under ischemic conditions by attenuating Ca^2+^ excitotoxicity and GSH depletion [70]. Another donor, MTC (a conjugate of S-allyl-L-cysteine and gallic acid), protected neurons from I/R injury by activating several antioxidant enzymes, including SOD, catalase (CAT), and glutathione peroxidase (GPx), and by reducing LDH levels. It activated phosphatidylinositol 3-kinase/protein kinase b (PI3K/AKT) and mitogen-activated protein kinase kinase/extracellular signal-regulated kinase (MEK/ERK) signaling, inhibited proapoptotic proteins, endoplasmic reticulum stress (ER) stress, two-pore domain potassium channel trek-1 (TREK-1), and inflammatory mediators, thereby promoting axonal regeneration and reducing neuronal damage (Figure 3, Table 2) [71].

Another promising H_2_S donor is N-acetylcysteine (NAC). In preclinical studies, NAC has repeatedly shown pronounced neuroprotective effects when administered within the first few hours after experimental TBI. For example, in a mouse model of severe TBI followed by secondary hypoxic insult, NAC started 2 h post-injury significantly reduced acute axonal damage and attenuated both early and delayed hippocampal neuron loss. However, NAC treatment did not affect the final cortical lesion volume and was not accompanied by improvement in long-term cognitive or behavioral outcomes compared with placebo-treated animals [72]. In addition, a Phase I randomized, double-blind, placebo-controlled trial in children aged 2–18 years with Glasgow Coma Scale ≤8 evaluated the safety and pharmacokinetics of the combination of N-acetylcysteine and probenecid administered enterally during the first days after injury. No serious adverse events related to the study treatment were recorded. Stable concentrations of both drugs were detected in cerebrospinal fluid for 72 h after treatment initiation. Nevertheless, no differences were found between the active-treatment and placebo groups in intracranial pressure, intensity of therapy, brain-injury biomarkers, or functional outcome at 3 months (Table 2) [73].

### 3.3. Natural H_2_S Donors

In addition to synthetic compounds, increasing attention is being paid to natural H_2_S donors, which provide gentle, thiol-dependent H_2_S release closely linked to endogenous pathways. Particular interest is focused on organosulfur metabolites from plants of the *Allium* (garlic, onion) and *Brassicaceae* (broccoli, cabbage) families, which serve as physiological reservoirs of H_2_S, which is slowly liberated under cellular conditions [74,75].

Thanks to their natural structural diversity and good bioavailability, these compounds exert pleiotropic effects on nervous tissue and demonstrate neuroprotective activity across a wide range of pathological conditions. Prolonged H_2_S release at physiological concentrations, excellent metabolic safety, and the ability to enhance the body’s own defense systems make natural organosulfur compounds not only promising nutraceutical agents but also an important tool for the prevention of neuropathologies and the maintenance of neuronal homeostasis.

DADS (diallyl disulfide), a garlic-derived allicin metabolite, reduced cerebral infarct volume by inhibiting astrocyte activation and pyroptosis mediated by the NLRP3/Caspase-1/IL-1β cascade, as well as by decreasing lactate dehydrogenase (LDH) expression and pro-inflammatory cytokines such as IL-1β and interleukin-18 (IL-18), thereby promoting regenerative processes [76]. In sciatic nerve injury models, DADS enhanced the anti-allodynic and anti-hyperalgesic effects of μ- and δ-opioid receptor agonists through upregulation of their expression in dorsal root ganglia (DRG). Additionally, it inhibited oxidative stress and apoptosis in the medial septum and DRG via H_2_S-dependent signaling pathways [77]. In oxidative stress-induced cataract models, DATS (diallyl trisulfide) restored GSH content and SOD activity while reducing LDH-mediated cytotoxicity (Table 3) [78].

Another natural H_2_S donor of interest is S-adenosylmethionine (SAMe), which exhibited even stronger neuroprotective effects than DADS in cuprizone-induced demyelination, effectively suppressing demyelinating pathology. Both H_2_S modulators reduced neuroinflammation, enhanced oligodendrocyte activity and autophagy via the AMPK/sirtuin 1/unc-51-like autophagy activating kinase 1/beclin 1 (AMPK/SIRT1/ULK1/beclin1) pathway, increased antioxidant levels (GSH and total antioxidant capacity (TAC)), and inhibited fibronectin aggregation as well as NF-κB and IL-17 expression (Table 3) [79].

Particular interest is focused on the natural isothiocyanate sulforaphane (SFN) from broccoli sprouts, which acts as a slow H_2_S donor. In a rat model of transient middle cerebral artery occlusion, pre-treatment with SFN markedly increased Nrf2 and HO-1 expression in cerebral microvessels and perivascular astrocytes, significantly reducing BBB disruption, infarct volume, and neurological deficit [80]. SFN also protected the brain in TBI models by decreasing edema and BBB permeability through Nrf2 pathway activation. When administered as early as 1 h post-TBI, SFN significantly improved spatial memory and working memory; however, the beneficial effect was completely lost if treatment was delayed to 6 h [81]. SFN prevented TBI-induced downregulation of AQP4 at the injury site, increased aquaporin-4 (AQP4) expression in the penumbra, and markedly reduced brain edema by enhancing water clearance through aquaporin channels [82]. A clinical trial (NCT04252261) is planned in which 90 patients with frontal lobe injuries will receive SFN or placebo for 12 weeks to assess its effects on cognitive functions. It is expected that SFN will reduce cognitive deficit and improve memory and learning (Table 3) [83].

### 3.4. Innovative Multicomponent Hybrid H_2_S Donors

Significant progress in the therapeutic application of H_2_S is demonstrated by innovative delivery systems that incorporate it into nanostructures, biomaterials, and adaptive polymer matrices. These platforms are forging a new direction in regenerative medicine, as they not only transport H_2_S to the target site but also ensure its local, prolonged, and physiologically relevant release synchronized with the microenvironment of damaged nervous tissue. Their architecture—ranging from nanoparticles and liposomes to functional hydrogels and metal–organic frameworks—enables precise control over release kinetics, bioavailability, tissue selectivity, and interaction with diverse cellular targets. A key advantage of such systems is their ability to combine transport, protective, and therapeutic functions in a single platform: nanoparticles can deliver H_2_S deep into the lesion while protecting it from premature degradation, and hydrogels create a stable biocompatible niche that supports regenerative processes [84]. It is precisely for this reason that innovative carriers have become the main tool for realizing the potential of H_2_S in the treatment of injuries to both the central and peripheral nervous systems. The use of these materials overcomes the limitations of traditional H_2_S donors—instability, systemic toxicity, and a narrow therapeutic window—providing targeted and long-term neuroprotective effects [85,86].

**Figure 4:**
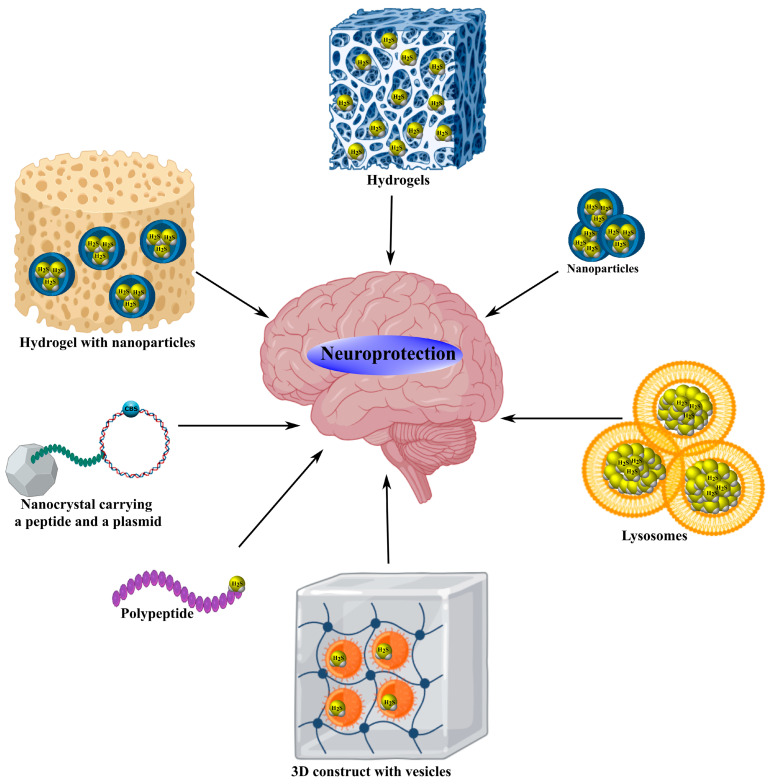
Neuroprotective mechanisms of hybrid H_2_S donors. Innovative hybrid H_2_S donors, including G16 MPG-ADT nanoparticles, ZnS NP, DATS@MION-PEG-LF, AP39@Lip liposomes, CdSe/ZnS nanocrystals with angiotensin-1 peptide and CSE plasmid, GelMA@LAMC, H_2_S@SF, SF-G@Mn, mPEG-PA-PP, MnS@AC hydrogels, PF-127@OMSN-JK hydrogel with nanoparticles, Zn-CA MOF, DATS-MSN, 3D/GelMA/EVs construct with vesicles, and SHI polypeptide, provide controlled H_2_S release, effectively suppressing apoptosis by inhibiting signaling pathways involving Bax, Bad, caspases, MLKL, and MAPK, reducing oxidative stress by decreasing MDA, NO, and iNOS, and attenuating neuroinflammation by blocking NLRP3, TNF-α, IL-1β, and NF-κB. These structures activate antioxidant systems, increasing levels of GSH, SOD, CAT, GPx, and Nrf2, modulate microglia via AMPK and PINK1/Parkin, stabilize BBB permeability, stimulate neuronal regeneration, angiogenesis, and cell migration through mTOR, STAT3, and Src/Fak/Pyk2, improving motor and cognitive functions in models of ischemia/reperfusion, neurotrauma, stroke, TBI, and Parkinson’s disease.

In a spinal cord injury model, G16 MPG-ADT nanoparticles (186.5 nm) carrying a specialized H_2_S-associated neuroprotective agent effectively released H_2_S, which promoted regeneration of damaged neurons via upregulation of mammalian target of rapamycin (mTOR) and signal transducer and activator of transcription 3 (STAT3) expression, resulting in improved motor recovery [87]. Similarly, zinc sulfide nanoparticles (ZnS NPs) in ischemic stroke models reduced infarct volume by continuously generating H_2_S, protecting neurons from apoptosis, stimulating neurovascularization through phosphorylated adenosine monophosphate-activated protein kinase (p-AMPK) modulation, and alleviating inflammatory responses [88]. Polyethylene glycol (PEG)- and lactoferrin (LF)-modified mesoporous iron oxide nanoparticles (MIONs) conjugated with diallyl trisulfide (DATS@MION-PEG-LF) provided sustained H_2_S release in the brain during ischemic injury. These nanoparticles exhibited excellent biocompatibility without eliciting cytotoxic or antigenic responses, while demonstrating antiapoptotic, anti-inflammatory, and antioxidant effects (Figure 4, Table 4) [89].

Liposomes encapsulating AP39 also exhibited remarkable H_2_S-dependent neuroprotective properties, including preservation of BBB integrity, reduction in oxidative stress, and mitigation of mitochondrial dysfunction [90]. Notably, cadmium selenide/zinc sulfide (CdSe/ZnS) nanocrystals conjugated with angiotensin-1 peptides were used to deliver CSE plasmids for localized H_2_S production in injured myocardial cells, leading to reduced infarct size, oxidative stress, and mitophagy via inhibition of the c/ebp homologous protein/glucose-regulated protein 78/eukaryotic initiation factor 2 alpha (CHOP/GRP78/eIF2α) signaling pathway (Figure 4, Table 4) [91].

Hydrogels capable of H_2_S release have also shown substantial therapeutic potential for neurotrauma treatment. For example, the composite hydrogel GelMA@LAMC, incorporating an H_2_S donor activated by ROS and integrated into a zinc–citrate metal–organic framework, inhibited oxidative stress, neuroinflammation, and mitochondrial dysfunction, while protecting neurons and stimulating angiogenesis after SCI [92]. A thermosensitive hydrogel pluronic f-127 (PF-127) containing ordered mesoporous silica nanoparticles (OMSN) loaded with JK (OMSN@JK), combined with stem cells was utilized for SCI therapy (Table 4). This hydrogel demonstrated excellent biocompatibility and neuroprotective efficacy by promoting neuronal differentiation and regeneration while suppressing neuroinflammatory processes [93].

A ferrofluid-based hydrogel containing Fe_3_S_4_ (FFH) reduced inflammatory factor expression through NF-κB pathway inhibition and participated in axonal remodeling, improving functional recovery after spinal cord injury [94]. Moreover, an H_2_S-associated silk fibroin hydrogel (H_2_S@SF) used in intracerebral hemorrhage models enabled sustained H_2_S release, which stabilized cerebral water homeostasis, reduced lesion area, and limited neuronal death in the striatum, cortex, and hippocampus [95]. H_2_S@SF also proved effective in TBI models by suppressing pyroptosis and necroptosis, alleviating edema, neuroinflammation, and neurodegenerative changes, and improving cognitive performance (Table 4) [96].

A related adaptive hydrogel based on a silk fibroin matrix, SF-G@Mn, provided gradual release of H_2_S, Mn^2+^, and bFGF, thereby modulating both acute and chronic posttraumatic stages of spinal cord injury. SF-G@Mn inhibited oxidative stress and neuroinflammation while promoting regenerative processes associated with axonal growth and myelin production (Figure 4, Table 4) [97].

In a recent study, a unique 3D-printed scaffold (3D/GelMA/EVs) based on gelatin methacryloyl (GelMA) hydrogel integrated with extracellular vesicles from H_2_S-preconditioned mesenchymal stromal cells was developed. This construct, tested in a spinal cord injury model, enhanced miR-7a-5p expression and improved motor function recovery [98]. In peripheral nerve transection models, a polymeric hydrogel mPEG-PA-PP containing an H_2_S donor localized within a ROS-sensitive polymer suppressed oxidative stress, inflammation, and mitochondrial dysfunction, thereby stimulating neuroregenerative processes (Table 4) [99].

A pH-sensitive polysaccharide hydrogel MnS@AC containing α-phase manganese sulfide nanoparticles (MnS NPs) inhibited inflammation while promoting proliferation and angiogenesis, with MnS NPs not affecting the pro-inflammatory cyclic gmp-amp synthase-stimulator of interferon genes (cGAS–STING) pathway (Figure 4, Table 4) [100].

A novel photoresponsive H_2_S donor for nerve regeneration has been proposed—an implantable Zn–CA metal–organic framework (MOF) matrix enabling controlled release of H_2_S and Zn^2+^. The combination of antioxidant and anti-inflammatory properties, together with angiogenesis stimulation and guided cellular migration, contributed to efficient neural and motor recovery [101]. In an I/R injury model, a thermosensitive polymer poly(n-isopropylacrylamide-co-n-tert-butylacrylamide) (PNNTBA) coating mesoporous silica nanoparticles conjugated with diallyl trisulfide (DATS-MSN) provided prolonged H_2_S release through its soluble polymeric shell, exerting cytoprotective effects [102]. Another hybrid H_2_S donor, consisting of MIONs loaded with DATS and enclosed within an erythrocyte membrane shell (RBC-DATS-MION), ensured effective and sustained H_2_S release associated with antiapoptotic activity (Table 4) [103].

Development of H_2_S-associated polypeptides represents another promising therapeutic direction for diverse pathological conditions, including neurotrauma. For instance, the designed insulin-derived polypeptide insulin-derived polypeptide (SHI), capable of donating H_2_S, reduced α-synuclein levels, increased dopamine transporter (DAT) expression, and improved behavioral outcomes in Drosophila and C. elegans Parkinson’s disease models (Figure 4, Table 4) [104].

The mitochondrial donor AP39 reduced infarct size, reactive microglial activation, and pro-inflammatory markers such as ionized calcium-binding adapter molecule 1 (Iba1), IL-1β, IL-6, and TNF-α, as well as the proneurotrophin growth factor-p75 neurotrophin receptor/sortilin/caspase-3 (proNGF–p75NTR–sortilin/caspase-3) axis, while inducing neuroprotective BDNF–tropomyosin receptor kinase b (BDNF–TrkB) and nerve growth factor-tropomyosin receptor kinase a (NGF–TrkA) signaling during cerebral ischemia [105]. In similar models, AP39 alleviated excitotoxicity by reducing glutamate and vesicular glutamate transporter 1 (VGLUT1) levels and upregulating glutamate transporter-1 (GLT-1) expression [106]. In a photothrombotic stroke model, AP39 decreased infarct volume and promoted mitophagy via activation of the PINK1/Parkin pathway, showing sex-dependent effects (Table 4) [107].

An improved formulation, AP39@Lip (liposome-integrated AP39), administered intranasally, provided targeted mitochondrial delivery in neurons during acute brain injury. AP39@Lip exhibited strong neuroprotective properties by mitigating mitochondrial dysfunction, oxidative stress, neuroinflammation, and apoptosis, with its antiapoptotic effects attributed to inhibition of ERK1/2 and caspase-1 activation (Figure 4) [108]. Another novel ROS-responsive H_2_S donor, HSD-R, demonstrated selective mitochondrial targeting, where it reduced apoptosis via inhibition of bh3-interacting domain death agonist/apoptotic protease activating factor-1/proapoptotic protein p53 (Bid/Apaf-1/p53) signaling, attenuated inflammation, and promoted angiogenesis (Table 4) [109].

Hybrid molecules based on SFN also attract interest: these are covalently linked constructs in which the isothiocyanate fragment of SFN is directly attached to the carboxylic group of classical NSAIDs, allowing the resulting compound to simultaneously potently and selectively inhibit COX-2, retain the anti-inflammatory activity of the original NSAID, and release protective H_2_S in tissues (Table 4) [110].

### 3.5. Inhibitors of H_2_S-Synthesizing Enzymes

Significant progress in modulating endogenous H_2_S in neurotrauma is associated with the use of inhibitors of its key biosynthetic enzymes: CBS, CSE, and 3-MST. However, in the majority of preclinical neurotrauma models, pharmacological suppression of H_2_S production predominantly exerts negative effects, exacerbating secondary injury, brain edema, cognitive deficits, and other damage [111]. Nevertheless, in conditions characterized by cytotoxic H_2_S concentrations, the use of these inhibitors opens new therapeutic prospects for treating various pathological states, including those associated with traumatic injury to the nervous system [112].

**Figure 5:**
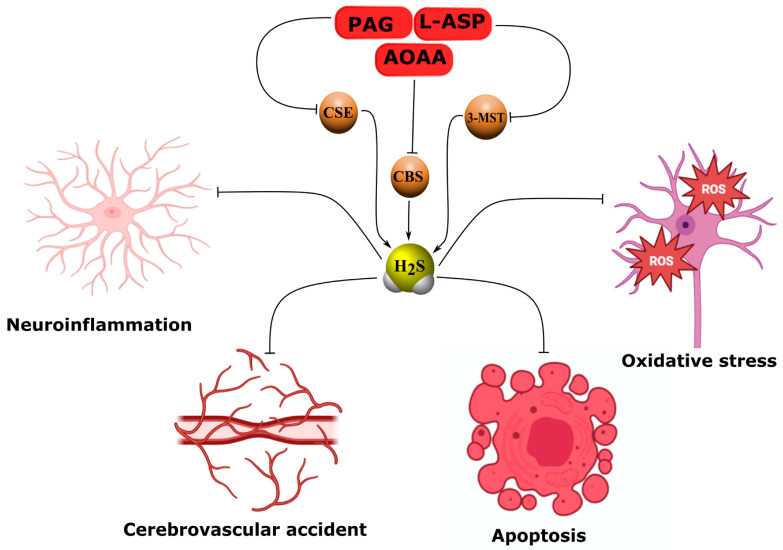
Neuropathological effects of H_2_S synthesis inhibitors. Inhibitors of H_2_S synthesis, including AOAA, PAG, oxamic hydrazide 1, L-aspartic acid, benserazide, and aurintricarboxylic acid, induce neuroinflammation, oxidative stress, apoptosis, and cerebrovascular spasm by blocking H_2_S production. AOAA, inhibiting CBS and MAS, reduces NAD^+^/NADH, STAT3, miR-9-5p, enhances expression of iNOS, COX2, TNF-α, IL-1β, CXCL11, p53, APP, reactive astrogliosis, and sympathetic activity, worsening neuroprotection in TBI, I/R, and hypoxia. PAG, blocking CSE, abolishes the effects of octreotide and remote preconditioning, reducing Nrf2, GSH, the KATP/BK channels, and vasodilation, while increasing oxidative stress and TNF-α. L-aspartic acid and oxamic hydrazide 1 suppress 3-MST and CSE, impairing vasodilation via RhoA-ROCK. Benserazide and aurintricarboxylic acid inhibit CBS and CSE, exacerbating pathological processes. Arrows with pointed ends indicate activation, while blunt ends indicate inhibition.

AOAA, an effective inhibitor of both the malate–aspartate shuttle (MAS) and CBS, reduced lipopolysaccharide-induced neuroinflammatory responses by suppressing reactive microglial activation and downregulating iNOS, COX-2, IL-1β, IL-6, and TNF-α expression both in vivo and in vitro. This effect was associated with a decrease in the cytosolic NAD^+^/NADH ratio and reduced STAT3 phosphorylation [113]. In a model of hypoxic–ischemic brain injury, AOAA inhibited L-cysteine-dependent neuroprotection by partially decreasing miR-9-5p and CBS expression and increasing pro-inflammatory cytokines such as TNF-α, IL-1β, and CXCL11 [114]. Likewise, in a cerebral ischemia/reperfusion model, AOAA blocked the neuroprotective effects of total rhododendron flavones (TFR), which act by inhibiting reactive astrogliosis and the rhoa/rho-associated protein kinase (RhoA/ROCK) signaling pathway [115]. Under conditions of retinal oxidative stress, AOAA did not affect the neuroprotective action of cannabinoids, whereas the 3-MST inhibitor α-ketobutyric acid abolished this effect [116]. It has been reported that AOAA completely suppressed brain H_2_S oscillations induced by spreading depolarization (SD) [117]. AOAA also reduced H_2_S production in the hypothalamus, thereby increasing arterial pressure and sympathetic activity of the urinary system in paroxysmal sympathetic hyperactivity [38]. In our own studies, AOAA administration exacerbated neuronal and glial cell death following TBI and upregulated proapoptotic proteins such as p53 [21], iNOS, and APP (Figure 5, Table 5) [20].

Similarly, propargylglycine (PAG), a selective inhibitor of CSE, abolished the neuroprotective effects of octreotide by inhibiting H_2_S generation and Nrf2 expression while increasing TNF-α levels in TBI [118]. In a vascular dementia model, both PAG and AOAA markedly suppressed the effects of remote preconditioning by reducing H_2_S, CBS, CSE, and Nrf2 expression and enhancing oxidative damage in nervous tissue [119]. PAG decreased H_2_S generation in cerebral vascular endothelium and reduced arteriole vasodilation, disrupting H_2_S-dependent activation of ATP-sensitive potassium (KATP) and large-conductance calcium-activated potassium (BK) channels [120]. Moreover, PAG rendered astrocytes carrying IDH1 mutations more vulnerable to cysteine deficiency, increasing oxidative stress and depleting GSH [121]. In cerebral arterioles, PAG significantly inhibited potassium-induced vasoconstriction through signaling pathways involving both H_2_S and eNOS/soluble guanylate cyclase (sGC) (Figure 5, Table 5) [122].

Additionally, oxamic hydrazide 1 has been identified as a promising selective inhibitor of H_2_S production due to its ability to bind to the pyridoxal phosphate (PLP) active site of CSE [123]. L-aspartic acid (L-ASP), an inhibitor of 3-MST, when combined with PAG, reduced acetylcholine-induced H_2_S generation and vasodilation in cerebral arteries, enhancing activation of the RhoA–ROCK pathway [124]. Benserazide, an inhibitor of CBS, in combination with paclitaxel, decreased SIRT1 sulfhydration and hypoxia-inducible factor 1-alpha/vascular endothelial growth factor (HIF-1α/VEGF) expression in tumor models [125]. Finally, aurintricarboxylic acid (NSC4056) demonstrated selective inhibition of CSE, effectively eliminating hypotension in a hemorrhagic shock model (Figure 5, Table 5) [126].

## 4. Discussion

The extensive preclinical data accumulated in global science on the biological effects of H_2_S modulators across diverse experimental models of neurotrauma indicate that H_2_S plays a fundamental role as an endogenous gasotransmitter capable of modulating key pathological processes. These include neurodegenerative changes in neurons and glial cells induced by oxidative stress, neuroinflammation, apoptosis, and other mechanisms [7,8,13,127,128,129,130]. Collectively, these data indicate the potential feasibility of integrating H_2_S-associated neuroprotective agents into the global healthcare system. However, this translational process faces several challenges, such as the lack of a unified concept of H_2_S-dependent neuroprotection and the heterogeneity of experimental data, which require comprehensive systematic analysis. Below, we discuss the key patterns identified through qualitative analysis of various classes of H_2_S donors, as well as inhibitors of its synthesis, with emphasis on their mechanisms of action, structural features, and potential therapeutic applications in neurotrauma.

Inorganic H_2_S donors, particularly fast-releasing ones such as NaHS, consistently demonstrate the ability to suppress oxidative stress and inflammation in models of TBI, where NaHS administration not only elevates H_2_S levels in nervous tissue and inhibits the expression of damage biomarkers but also promotes the restoration of synaptic plasticity by increasing the expression of BDNF, ARC, and PSD-95 [27], as well as regulating NMDA receptor activity and levels of glutamate, Ca^2+^, and microtubule-associated proteins such as CaMKII [26]. These changes correlate with improvements in cognitive function [26,27]. Microinjections of NaHS into the hypothalamus restore disulfide bonds in NMDAR1 (Table 6) [38].

Synaptic plasticity impairment is an inseparable feature of both the brain [131] and SCI [132], severely limiting neuronal regeneration. BDNF plays a key role as a regulator of neuronal survival, promoting regeneration and recovery of synaptic transmission after traumatic insult [133]. Reduced ARC expression is associated with neurodegenerative processes and the induction of endoplasmic reticulum stress and necroptosis following TBI [134]. PSD-95, a major structural component of the postsynaptic density, ensures synaptic organization, with its expression and phosphorylation forming a dynamic system under neuronal stress (Table 6) [135].

All of these proteins are functionally linked through NMDA receptor-mediated signaling pathways, which represent central nodes of neurotoxicity following TBI by promoting oxidative stress and inflammation through mechanisms of excessive sodium and calcium influx, disrupting neuronal homeostasis, and triggering subsequent cell death [136]. Interestingly, H_2_S can directly interact with NMDA receptor subunits, modulating their activity and influencing synaptic dynamics and plasticity (Table 6) [137].

The neuroprotective effects of NaHS are also evident in controlled cortical impact models, where NaHS activates mitoK_ATP and antioxidant systems, reducing brain edema, BBB permeability, and lesion size [28]. Mitochondrial dysfunction is known to play a pivotal role in propagating secondary injury following TBI, initiating molecular and cellular cascades that culminate in cell death [138]. Activation of mitoK_ATP channels inhibits aggressive free radical processes and inflammation [139], mitigating TBI-induced cognitive impairments through multiple neuroprotective signaling pathways [140]. These channels are essential for maintaining mitochondrial integrity and function. During ischemia, mitoK_ATP channels sustain membrane transport, prevent ATP influx into the mitochondrial matrix, and inhibit mPTP opening [141]. H_2_S can modulate mitoK channel activity directly or indirectly through regulation of various signaling pathways or via S-sulfhydration (Table 6) [142].

Interestingly, NaHS can also exert neuroprotective effects in models of peripheral nerve injury by reducing dopaminergic neuron loss in the midbrain through inhibition of IL-17–mediated pathways involving MLKL and tyrosine hydroxylase [29] and by modulating glutamatergic transmission via astrocytes and EAAT2 in the spinal cord [30]. It also attenuates degeneration in dorsal root ganglia and axons through activation of SOD and SOD2 and suppression of aldose reductase (Table 6) [31].

TBI induces profound alterations in the dopaminergic system, including disrupted dopamine levels, disconnection of projection pathways, and altered expression of the dopamine transporter (DAT) and dopamine receptor D2 (D2R) in different brain regions, thereby impeding neuronal network recovery and increasing the risk of long-term consequences [143]. It has been reported that H_2_S may induce S-sulfhydration of AMP-activated protein kinase (AMPK), enhancing its phosphorylation and promoting autophagy in dopaminergic cells [144]. Moreover, H_2_S elevates dopamine levels in C. elegans, reducing oxidative stress [145]. The combined NO/H_2_S-releasing drug NOSH-aspirin reduces phosphorylation of JNK, p38, and ERK, providing cytoprotection to dopaminergic neurons in Parkinson’s disease models [146]. Studies also show that H_2_S prevents the death of tyrosine hydroxylase–positive neurons in the substantia nigra through activation of mitoK_ATP channels [147]. Additionally, dopamine D1 receptors (DR1) can modulate H_2_S levels via activation of CSE, decreasing apoptosis of vascular endothelial cells [148]. Reduction in oxidative stress by H_2_S may occur both through direct scavenging of ROS and reactive radicals [149] and indirectly through the activation of antioxidant defense systems, including GSH, thioredoxin (Trx-1), CAT, SOD, and GPx (Table 6) [10].

In cerebral I/R models, NaHS inhibits ROCK2 via specific phosphorylation at Thr436 and Ser575, reducing LDH, NSE, Bax, and oxidative stress while increasing Bcl-2 expression and cognitive performance [32]. Similarly, in hippocampal neuronal cultures, NaHS suppresses phosphorylation of ROCK2, PLK1, and PKA, thereby enhancing cell survival under hypoxia/reoxygenation conditions [33]. ROCK2 is broadly expressed in the brain and spinal cord, regulating various signaling pathways responsible for migration, phagocytosis, cytokine release, and other functions that ultimately determine neuronal fate under traumatic stress [150]. Suppression of CBS expression has been shown to reduce ROCK2 levels and reactive astrocyte proliferation under I/R conditions [151]. Furthermore, H_2_S may promote RhoA phosphorylation at Ser188, inhibiting the RhoA/ROCK signaling pathway and exerting neuroprotective effects (Table 6) [152,153].

Moreover, in models of cardiac arrest [34] and lateral percussion-induced TBI [35,36], NaHS stabilizes the BBB by inhibiting MMP-9 and occludin [34], prevents vascular dysfunction, hypertension, and sympathetic hyperactivity, and restores CBS, CSE, and eNOS expression [35,36]. It has been reported that H_2_S can regulate MMP levels through transcription-dependent mechanisms as well as S-sulfhydration [154]. Given that MMP-9 inhibitors are considered promising neuroprotective agents—since this enzyme exacerbates trauma-induced BBB disruption by degrading microvascular basement membranes and tight junctions [155,156]—H_2_S-mediated regulation of MMPs represents a crucial therapeutic mechanism. Occludin, in turn, is one of the major BBB proteins ensuring its structural and functional integrity [157]. Numerous studies using diverse experimental models confirm the role of H_2_S in BBB stabilization (Table 6) [158,159].

Transitioning to other inorganic donors, such as Na_2_S, a similar but more mediated dynamic is observed. For instance, in models of intracerebral hemorrhage, Na_2_S inhibits neurotoxicity, axonal degradation, and inflammation, surpassing Na_2_S_3_ in preventing neuronal death [40]. In global ischemia/reperfusion (I/R), Na_2_S persulfidates caspase-3 at Cys163 via the intermediate metabolite sodium thiosulfate (STS), transported by SLC13A4 [41]. These findings align with our previous data on reduced neuronal and glial cell death in TBI and axotomy through suppression of p53, with enhanced apoptosis upon inhibition of CBS by AOAA [19], as well as colocalization of p53 with fragmented nuclei [21] and inhibition of iNOS and APP [20]. Notably, p53, also known as a tumor suppressor or “guardian of the genome,” is a pivotal protein in neuronal survival and death, regulating numerous intracellular functions, including cell cycle, metabolism, proliferation, DNA repair, and apoptosis. Elevated p53 levels in neurons and glial cells during neurotrauma are typically associated with activation of proapoptotic signaling [160,161]. It is established that H_2_S can modulate p53 through mechanisms that reduce oxidative stress, activate antioxidant defense systems, and persulfidate cysteine residues of p53, altering its conformation and functional activity [162]. In turn, APP, widely recognized as a key molecular player in Alzheimer’s disease pathogenesis, is also a critical factor in various physiological processes, ranging from cellular differentiation to complex cascades determining cell fate. APP expression rapidly increases in TBI, triggering cytotoxic cascades via its full-length form or active proteolytic products [163]. H_2_S has been shown to regulate APP and its cleavage products through persulfidation of cysteine residues [164]. Additionally, iNOS, whose expression in neurotrauma leads to cytotoxic NO concentrations, can be targeted by H_2_S through inhibition of its transcription factor NF-κB (Table 6) [165].

STS further amplifies this pattern by increasing H_2_S and GSH levels in glial cells, suppressing TNF-α, IL-6, p38 MAPK, and NF-κB in a dose- and time-dependent manner [42], stimulating VEGF-mediated angiogenesis in ischemia [44], and, in our studies, demonstrating pH-dependent modulation of p53 via weak van der Waals interactions with Arg248, inducing conformational fluctuations without stable complex formation. This acts as a metabolic sensor in acidotic TBI zones, reducing apoptosis and enhancing neuronal survival [21]. TBI is known to induce significant metabolic disturbances, leading to a critical pH drop in the injury zone, which causes conformational changes in proteins [166]. Using in silico methods, we found that at physiologically normal pH, H_2_S, predominantly in the form of HS^−^, does not interact with p53. However, at a pH of ~6.5, H_2_S concentration increases, enabling interaction with p53 at the Arg248 site [21]. H_2_S also regulates another key transcription factor, NF-κB, which negatively impacts neuronal and glial cell survival, through modulation of signaling pathways such as p38MAPK/mTOR [167] and SIRT1/mTOR [168] in traumatic injury. Interestingly, H_2_S has been reported to enhance NF-κB activity by persulfidating cysteine-38 of the p65 subunit, increasing its DNA binding and interaction with the coactivator RPS3, a process further amplified by TNF-α (Table 6) [169].

Thiol-activated donors, such as COS/H_2_S and AlaCOS, are of significant interest for research and medical applications. These compounds enable tracking of H_2_S release using fluorophores [45,46] and facilitate tissue-specific H_2_S delivery [47]. This opens new prospects for developing hybrid systems and advancing approaches to controlled-release drug design, a critical step toward precision therapy (Table 6).

In contrast, organic H_2_S donors, such as GYY4137, are characterized by slow release. In spinal cord I/R models, GYY4137 inhibits Bax, Bad, caspases, MLKL, p-RIP1/3, NLRP3, and pro-inflammatory factors [50], stabilizing the BBB by suppressing autophagic degradation of occludin [51] and sympathetic transmission via K_ATP channels and prostanoids. This aligns with rapid donors in antioxidant mechanisms but offers prolonged protection [53]. Notably, the prolonged release of H_2_S is a significant advantage of organic donors, as high H_2_S concentrations can exert cytotoxic effects, leading to structural and functional impairments in neural cells by inhibiting cytochrome c oxidase, reducing mitochondrial potential, and enhancing free radical processes (Table 6) [170].

ACS67 mitigates electroretinogram changes in retinal ischemia, increasing GSH [56] and suppressing norepinephrine via K_ATP [53]. Similarly, ACS84 outperforms NaHS in inhibiting lipid peroxidation, MAO-B [57], and mitochondrial dysfunction in Parkinson’s disease, stimulating Nrf2 and suppressing NO [58], TNF-α, and p38/JNK-MAPK in Aβ-induced microglia [59]. SPRC (ZYZ-802), by modulating CBS, reduces astrogliosis, Aβ deposition, and inflammation in Alzheimer’s disease via NF-κB/MAPK, inhibiting TNF-α [60], COX-2, and ERK1/2 [61], and enhancing microglial migration through CD24 and Src/Fak/Pyk2 [62], effects comparable to those in stroke and neuroinflammation [63]. These findings suggest a consistent role for endogenous H_2_S stimulation in treating chronic neurodegenerative and inflammatory conditions. Although Alzheimer’s disease-related pathological changes differ from TBI, common patterns, such as brain atrophy and axonal degeneration, are observed [171]. Moreover, many molecular-cellular mechanisms in ischemia overlap with Alzheimer’s disease, including accumulation of misfolded proteins like Aβ and tau, activation of reactive astrocytes, impaired APP proteolysis, and BBB permeability [172], allowing for limited extrapolation of Aβ neurotoxicity model results to neurotrauma pathogenesis. Similarly, Parkinson’s disease shares molecular-cellular patterns with traumatic injury processes (Table 6) [173]. Thus, H_2_S acts as a versatile gasotransmitter, integrating multiple signaling pathways into a unified neuroprotective mechanism dependent on localization, timing, and concentration.

ADT-OH and derivatives, such as ADT and ATB-346, contribute to regeneration by modulating neural progenitor cell differentiation via β-catenin/TCF7L2 [64], reducing scarring and microglial activation in SCI [65], shifting microglia to the M2 phenotype via AMPK [66], and stabilizing the BBB through NF-κB in stroke [67], while suppressing Akt/VEGF/MMP9 [68] and edema in TBI [69]. This reveals similarities with inorganic donors in vascular protection but with an emphasis on regenerative processes. Modern organic donors, such as S-memantine and MTC, reduce Ca^2+^ excitotoxicity and GSH deficiency in ischemia [70], activate SOD, CAT, GPx, PI3K/AKT, and MEK-ERK, and inhibit apoptosis, endoplasmic reticulum stress, and TREK-1 [71], demonstrating targeted mechanisms for neuronal regeneration [70,71]. Notably, memantine, an NMDA receptor inhibitor, enhances dendritic branching, synaptic plasticity, and reduces neuroinflammation [174] making its use as an H_2_S donor particularly promising in traumatic injury by targeting a key node in neurotrauma pathogenesis, namely NMDA receptors (Table 6).

NAC is one of the most extensively studied candidate neuroprotective agents in TBI due to its potent antioxidant action, ability to replenish glutathione, modulation of neuroinflammation and ferroptosis, activation of the Nrf2-ARE pathway and several neurotransmitter systems, and its role as an H_2_S donor [175,176]. Preclinical models consistently demonstrate reduced acute axonal injury and long-term hippocampal neuron loss when NAC is administered within the first few hours post-injury; however, these morphological benefits are not accompanied by improved cognitive-behavioral outcomes and, most importantly, have been almost impossible to reproduce in clinical trials [72]. The key problem is poor NAC penetration through both the intact and, especially, the damaged blood–brain barrier, combined with active efflux via organic acid transporters. To overcome this limitation, a combination of NAC with probenecid (a classic inhibitor of organic anion transporters) was developed and tested in a Phase I pilot study (Pro-NAC, NCT01322009). The combination proved safe in children with severe TBI and provided stable concentrations of both drugs in cerebrospinal fluid for 72 h, but it did not result in differences in intracranial pressure, intensity of therapy, or functional outcome at 3 months [73]. Despite its broad spectrum of neurobiological effects and convincing preclinical data, the clinical efficacy of NAC in TBI remains unproven. The main barriers—late initiation of therapy in real-world practice and, most likely, still insufficient brain parenchymal concentrations—require further studies with earlier administration, alternative delivery routes, or new adjuvants to enhance BBB penetration (Table 6) [73,176].

Natural donors, such as DADS, reduce infarction by inhibiting astrogliosis and pyroptosis [76], enhancing opioid effects in sciatic nerve injury, and suppressing stress in dorsal root ganglia [77]. Similarly, DATS increases GSH/SOD under oxidative stress [78]. SAMe outperforms DADS in suppressing demyelination via AMPK/SIRT1/ULK1/beclin1, enhancing oligodendrocytes, autophagy, and antioxidants while inhibiting NF-κB/IL-17 [79]. Natural compounds offer a viable alternative to synthetic ones, exhibiting pronounced neuroprotective effects. Their ability to suppress pyroptosis, a critical process in CNS injuries [177], and activate autophagy as a mechanism for clearing damaged organelles and proteins [178] holds significant promise for safe clinical applications as natural compounds. It is worth noting that organosulfur compounds obtained from food exhibit a wide range of cytoprotective effects and can be used for the prevention of various diseases. At the same time, their practical application is limited by high chemical instability: the structure of organosulfides is easily altered by temperature, pH, and cooking methods. When garlic is crushed, alliin is converted by alliinase into unstable allicin, which rapidly decomposes to form oil-soluble polysulfides—primarily DADS and DATS [74]. It is these relatively stable polysulfides that serve as the main H_2_S donors in garlic preparations and garlic oil, providing slow physiological H_2_S release in tissues. Notably, DATS significantly outperforms DADS in H_2_S-donating efficiency: according to density functional theory calculations, nucleophilic attack by thiolate anions (Cys^−^ and GSH^−^) on the peripheral sulfur of DATS is both kinetically and thermodynamically more favorable than on DADS, leading to rapid formation of the allylperthiolate anion (ASS^−^) and subsequent H_2_S release. In the case of DADS, the analogous process is substantially slower and has a higher energy barrier, making it a much weaker donor compared to DATS. Thus, the majority of physiologically relevant H_2_S in garlic-derived preparations is generated predominantly from DATS in the presence of biological thiols (Table 6) [179].

At the same time, SAMe, a physiological methyl donor and precursor of the transsulfuration pathway, is actively investigated in both clinical practice and preclinical TBI models. By replenishing cysteine and glutathione stores, SAMe indirectly enhances endogenous H_2_S synthesis via CBS and CSE. The combination of SAMe with sulfur donors represents a promising strategy for synergistically increasing brain levels of both H_2_S and glutathione in the post-traumatic period, when methylation and redox processes are disrupted (Table 6) [180].

Particular interest is drawn to SFN, which acts as a slow H_2_S donor and a potent activator of the Nrf2 pathway. In preclinical models of ischemia [80] and TBI [81,82], SFN significantly reduced blood–brain barrier disruption and brain edema, restored AQP4 levels, decreased infarct size and neurological deficit, and, when administered within the first hours after TBI, markedly improved spatial and working memory. The neuroprotective potential of SFN is planned to be evaluated in the clinical trial NCT04252261, where its biological effects will be assessed over 12 weeks in patients with frontal lobe injury [83]. The cytoprotective action of SFN is realized primarily through activation of the key regulator of antioxidant defense—the Nrf2-ARE pathway—while SFN also functions as an H_2_S donor, triggering H_2_S-dependent signaling cascades that provide anti-inflammatory, vasorelaxant, and antiapoptotic effects. H_2_S release from SFN occurs slowly and in a thiol-dependent manner, making it physiologically preferable to fast synthetic H_2_S donors. Thanks to this dual activity—Nrf2 activation and H_2_S release—SFN exhibits pronounced cytoprotective properties and has facilitated the development of a series of H_2_S-releasing hybrid molecules that combine the molecular scaffold of NSAIDs with the isothiocyanate fragment of SFN, thereby liberating physiological levels of H_2_S (Table 6) [110].

Hybrid H_2_S donors represent a logical evolution in the paradigm of H_2_S-associated neuroprotective drugs, integrating advances in nanotechnology and biomaterial development for efficient delivery and release of the target molecular agent. Nanoparticles, such as G16 MPG-ADT, ZnS NP, and DATS@MION-PEG-LF, provide prolonged H_2_S release, stimulating regeneration via mTOR/STAT3 [87], p-AMPK, and neurovascularization while reducing apoptosis [88,89]. Similarly, liposomes with AP39 [90] and CdSe/ZnS nanocrystals delivering CSE plasmids for local H_2_S production [91] exhibit H_2_S-dependent neuroprotective effects. Nanoparticles are excellent molecular platforms for targeted drug delivery due to their large surface area and high penetration capacity [181]. They are widely used in preclinical studies across various experimental models, demonstrating a broad range of cytoprotective effects with high biocompatibility [182,183]. Liposomal carriers also provide an effective solution for molecular agent transport, offering excellent preservation, rapid biodegradability, and superior biocompatibility compared to nanoparticles, which may accumulate and cause cytotoxic effects (Table 6) [184,185].

Hydrogels, such as GelMA@LAMC, PF-127 with OMSF@JK, FFH with Fe_3_S_4_, H_2_S@SF, and SF-G@Mn, demonstrate adaptability by inhibiting stress and inflammation via NF-κB suppression, stimulating angiogenesis, differentiation, and myelination with prolonged release of H_2_S, Mn^2+^, and bFGF. These are particularly effective in spinal cord injury and TBI, suppressing pyroptosis/necrosis and stabilizing homeostasis [92,93,94,95,96,97]. ROS/pH-sensitive hydrogels, such as mPEG-PA-PP and MnS@AC, exert anti-inflammatory effects and promote proliferation and angiogenesis [99,100]. Similarly, 3D-printed scaffolds like 3D/GelMA/EVs with H_2_S-preconditioned vesicles provide protective effects by upregulating miR-7a-5p for motor recovery [98]. Hydrogels represent a further step toward effective neuroprotectors, serving as drug carriers with prolonged action and as matrices mimicking the extracellular matrix for transplanted cells, filling damaged cavities (Table 6) [186].

Photosensitive matrices, such as Zn-CA MOF, and thermosensitive PNNTBA with DATS-MSN, as well as RBC-DATS-MION, enable controlled release for antioxidant and antiapoptotic protection, highlighting a trend toward personalized delivery [101,102,103]. These donors allow precise control of H_2_S release through light and thermal regulation, unveiling new methods for modulating H_2_S concentration in injury zones (Table 6).

Polypeptides, such as SHI and mitochondrial donors AP39/HSD-R, reduce α-synuclein [104], stimulate BDNF-TrkB [105], suppress excitotoxicity [106], and mitophagy [107]. Liposomal AP39@Lip targets mitochondria, blocking ERK1/2/caspase-1 [108]. Finally, H_2_S synthesis inhibitors, such as AOAA, confirm these mechanisms by blocking neuroprotection in hypoxia/I/R, enhancing neuroinflammation [114], astrogliosis, and RhoA/ROCK [115], and suppressing H_2_S fluctuations during depolarization [117]. Similarly, PAG, by inhibiting H_2_S generation, increases pro-inflammatory cytokine levels and oxidative stress [118], abolishing the effects of octreotide and preconditioning [119], rendering cells vulnerable to cysteine deficiency [121], and inhibiting vasoconstriction/vasodilation (Table 6) [122].

Selective inhibitors, such as oxamic hydrazide 1 [123], L-aspartic acid [124], benserazide [125], and NSC4056 [126], underscore the potential for precise CSE/CBS inhibition to study H_2_S-dependent pathways, such as PLP binding, RhoA-ROCK, and SIRT1 persulfidation (Table 6).

However, inhibitors of H_2_S biosynthesis may also be employed for neuroprotection in conditions associated with cytotoxic elevations of H_2_S. Excessive administration of H_2_S donors or the generation of disproportionately high concentrations of exogenous H_2_S can be no less harmful than its deficiency.

For a long time, physiological sulfide concentrations in mammalian neural tissue were believed to range from 50 to 160 μM [187], with free H_2_S in tissues estimated at 30–100 μM. However, modern high-sensitivity analytical methods have revealed that the actual concentration of free, unbound H_2_S in the brain is only 10–30 nM [188], while the majority of sulfur exists in acid-labile and bound sulfane pools [189]. In neurodegenerative diseases and acute brain injuries, these levels are typically markedly reduced [10,18].

Conversely, excessive elevations of free H_2_S can shift its actions from protective to toxic [190]. Supraphysiological H_2_S induces oxidative stress by inhibiting antioxidant enzymes, enhancing ROS accumulation, and increasing the expression of pro-inflammatory mediators [10,12,17]. It also triggers apoptosis [191], directly inhibits cytochrome-c oxidase [192], and exerts additional detrimental effects.

Thus, both deficiency and excess of H_2_S can aggravate neurodegenerative processes and acute brain injury. These findings underscore the critical importance of precise control over H_2_S dosage, release kinetics, and tissue localization, the development of controlled and trigger-responsive delivery systems, and the need for further clinical studies to ensure the safe application of H_2_S-modulating strategies in neuroprotective therapy.

## 5. Conclusions

Overall, the analysis reveals common patterns: all classes of H_2_S donors suppress oxidative stress, inflammation, and apoptosis, stabilize the BBB, and stimulate regeneration, but differ in release rate, targeting, and effect duration. Inorganic donors provide rapid effects, organic donors offer prolonged action, natural donors ensure biocompatibility, and hybrid donors enable controlled and personalized delivery. Inhibitors confirm the role of H_2_S in pathogenesis, emphasizing the need for balance. Modulating H_2_S levels may be a promising therapeutic approach for neurotrauma. However, further clinical studies are needed to identify the optimal route of administration and reduce the risk of side effects. Study of this problem will contribute to further development of clinical neurotraumatology.

## Figures and Tables

**Table 2:** Neuroprotective effects of organic synthetic H_2_S donors in experimental models of central and peripheral nervous system injury. Arrows ↑ and ↓ denote increase and decrease, respectively. Abbreviations. Main active substance: H_2_S—hydrogen sulfide. Synthetic and hybrid H_2_S donors: GYY4137—morpholinium phosphorodithioate derivative; SPRC/ZYZ-802—S-propargyl-L-cysteine; ACS67—latanoprost-H_2_S hybrid; ACS84—L-DOPA-H_2_S hybrid; ATB-346—naproxen-H_2_S hybrid; S-memantine—memantine-H_2_S hybrid; ADT-OH/ADT—5-(4-hydroxyphenyl)-3H-1,2-dithiole-3-thione; MTC—S-allyl-L-cysteine-gallic acid conjugate; NAC—N-acetyl-L-cysteine. Main injury models: TBI—traumatic brain injury; BBB—blood–brain barrier; I/R—ischemia/reperfusion; CPR—cardiopulmonary resuscitation; CA/CPR—cardiac arrest/cardiopulmonary resuscitation; MCAO—middle cerebral artery occlusion; BCAO—bilateral carotid artery occlusion; SCI—spinal cord injury; MPTP—1-methyl-4-phenyl-1,2,3,6-tetrahydropyridine; 6-OHDA—6-hydroxydopamine; NMDA—N-methyl-D-aspartate; OGD—oxygen-glucose deprivation; Aβ—beta-amyloid; APP/PS1—transgenic Alzheimer’s disease mouse model; IOP—intraocular pressure. Key molecular targets and signaling pathways: K_ATP channels—ATP-sensitive potassium channels; MAPK—mitogen-activated protein kinases (p38 MAPK; ERK1/2; JNK); NF-κB—nuclear factor kappa B; PI3K/Akt—phosphatidylinositol-3-kinase/Akt; MEK-ERK—mitogen-activated/extracellular signal-regulated kinase; AMPK—AMP-activated protein kinase; CaMKKβ—calcium/calmodulin-dependent protein kinase kinase β; LKB1—liver kinase B1; Nrf2—nuclear factor erythroid 2-related factor 2; HO-1—heme oxygenase-1; GCLC/GCLM—glutamate-cysteine ligase subunits. Enzymes of H_2_S synthesis and inhibitors: CBS—cystathionine-β-synthase; AOAA—aminooxyacetic acid. Apoptosis and necroptosis protein markers: Bax—Bcl-2-associated X protein; Bad—Bcl-2-associated death promoter; Bcl-2—B-cell lymphoma 2; p-RIP1/3—phosphorylated RIP1/RIP3; MLKL—mixed lineage kinase domain-like protein; NLRP3—NLRP3 inflammasome; PARP—poly(ADP-ribose) polymerase; TUNEL—terminal deoxynucleotidyl transferase dUTP nick end labeling. Inflammatory and oxidative stress markers: TNF-α—tumor necrosis factor alpha; IL-1β—interleukin-1β; IL-6—interleukin-6; IL-10—interleukin-10; COX-2—cyclooxygenase-2; iNOS—inducible NO synthase; nNOS—neuronal NO synthase; NO—nitric oxide; NOX4—NADPH oxidase 4; ROS—reactive oxygen species; 8-OHdG—8-hydroxy-2′-deoxyguanosine; MDA—malondialdehyde. Antioxidant systems: SOD—superoxide dismutase; CAT—catalase; GPx—glutathione peroxidase; GSH—reduced glutathione. Neurotrophic and synaptic proteins: BDNF—brain-derived neurotrophic factor; TH—tyrosine hydroxylase; NF-L—neurofilament light chain; Thy-1—thymocyte antigen-1; synaptophysin—synaptic protein. BBB and tight junction proteins: occludin—tight junction protein; MMP-9—matrix metalloproteinase-9; VEGF—vascular endothelial growth factor. Other proteins and markers: GFAP—glial fibrillary acidic protein; Arg1—arginase-1; YM1—chitinase-3-like protein 3; CD24—cluster of differentiation 24; Src/Fak/Pyk2—microglia migration signaling pathway; β-catenin—beta-catenin; TCF7L2—transcription factor 7-like 2; c-Myc—oncogene c-Myc; Ngn1/2—neurogenin 1/2; α-synuclein—alpha-synuclein; APP—amyloid precursor protein; tPA—tissue plasminogen activator. Cell lines: SH-SY5Y—human neuroblastoma cell line; PC12—rat pheochromocytoma cell line; RGC-5—retinal ganglion cell line; BV2—mouse microglial cell line. Tests and assessment methods: MWM—Morris water maze; NOR—novel object recognition; ERG—electroretinography; LDH—lactate dehydrogenase; MTT—3-(4,5-dimethylthiazol-2-yl)-2,5-diphenyltetrazolium bromide cell viability assay.

Donor	Model, Animals, Concentration/Dose	Main Effects	Reference
GYY4137	Spinal cord I/R, rats, 50 mg/kg 30 min before reperfusion	↓ neuronal loss, inhibition of Bax, Bad, caspase-MLKL, p-RIP1/3, NLRP3, pro-inflammatory factors; stabilization of BBB, ↓ neuroinflammation, prevention of Nissl body degradation.	[50]
GYY4137	Cardiac arrest/CPR (global cerebral I/R), mice, 4 mg/kg i.p. 1 h after CA/CPR	↓ systemic inflammation, improved neurological outcome; protection of BBB, ↑ occludin content by inhibiting its autophagy-mediated degradation.	[51]
GYY4137	I/R, rats, 5 µL 1 mM into lateral ventricle before injury	↓ infarct volume and brain edema, improved neurological function (Garcia scale); ↑ p-p38 MAPK, p-ERK1/2, p-JNK; ↓ Bax, ↑ Bcl-2, ↓ caspase-3 activity (inhibition of apoptosis via MAPK pathway regulation).	[52]
GYY4137, L-cysteine, ACS67	Isolated bovine iris-ciliary bodies, 1–30 µM (GYY4137), L-cysteine (10 nM–10 µM), ACS67 (10 nM–10 µM)	Inhibition of sympathetic neurotransmission, suppression of [^3^H]-norepinephrine release via H_2_S, prostanoids, and K_ATP channels.	[53]
GYY4137	NMDA-induced retinal injury, mice, intravitreal 1, 10 and 100 nmol/eye (effective from 1 nmol)	Dose-dependent protection of retinal ganglion cells; ↓ TUNEL^+^ cells, ↓ 8-OHdG (antioxidant and antiapoptotic effect).	[54]
GYY4137	Parkinson’s disease model (MPTP, C57BL/6J mice), 50 mg/kg/day i.p. (effective dose); 12.5 and 25 mg/kg weaker	Improved motor functions (rotarod, beam, grid); protection of TH^+^ neurons in substantia nigra and striatum; ↓ nitrative stress, ↓ NO, ↓ nNOS (via Akt), ↓ α-synuclein nitration.	[55]
ACS67	Retinal I/R (IOP 120 mmHg for 50 min, rats) + in vitro (RGC-5 + H_2_O_2_); in vivo: 4 nM (5 µL intravitreal, single dose immediately after ischemia); in vitro: 1–50 µM	In vivo: protection of ERG (a- and b-waves), ↑ NF-L, Thy-1, ↓ PARP, GFAP, nNOS; protection of optic nerve axons. In vitro: ↑ GSH, ↓ ROS, ↓ apoptosis, protection from H_2_O_2_ toxicity (latanoprost alone does not protect).	[56]
ACS84	Parkinson’s disease model (6-OHDA); in vitro: SH-SY5Y + 6-OHDA (0.1 nM–10 µM); in vivo: rats, unilateral striatal 6-OHDA lesion, 10 mg/kg/day oral (gavage) for 3 weeks	In vitro: ↑ viability, ↓ LDH, ↓ ROS, ↑ SOD, nuclear translocation of Nrf2 → ↑ GCLC, GCLM, HO-1. In vivo: ↓ apomorphine-induced rotations, protection of TH^+^ neurons in SN, ↑ striatal dopamine, ↓ MDA, ↓ oxidative stress (L-DOPA and NaHS alone significantly weaker).	[57,58]
ACS84	Aβ-induced microglia, 10 µM	Inhibition of Aβ_1–40_ cytotoxicity via ↓ NO, TNF-α, p38- and JNK-MAPK; correction of mitochondrial dysfunction.	[59]
SPRC (ZYZ-802)	Alzheimer’s disease, APP/PS1 mice (12 mo), 100 and 200 mg/kg/day oral, 3–6 months; primary astrocytes + Aβ_1–40_ Astrocyte/neuron co-culture, 1–100 µM	↑ cognitive functions (MWM, NOR), ↓ astrogliosis (GFAP), ↓ dark neurons, ↓ lipofuscin ↓ insoluble Aβ deposition, ↓ Aβ_42_/Aβ_40_ ratio ↓ neuroinflammation (↓ p-NF-κB, p-MAPK, ↓ TNF-α, NO in astrocytes), ↑ CBS activity and expression, ↑ H_2_S in brain and plasma, protection of synaptophysin and neuronal survival in co-culture (effects abolished by CBS siRNA and AOAA).	[60]
SPRC	Aβ_25–35_ injection, rats, icv 10 µg, 40 and 80 mg/kg/day i.p., 7–14 days	↑ cognitive functions (water maze), protection of hippocampal neuron ultrastructure (↓ dark neurons, vacuolization), ↓ TNF-α, COX-2 mRNA, ↓ p-ERK1/2, ↓ IκB-α degradation, ↓ NF-κB activation.	[61]
SPRC	Ischemic stroke (mice, MCAO 60 min + reperfusion), 10 mg/kg i.p. immediately after MCAO + every 12 h × 3	↑ CD24 via CBS/H_2_S, inhibition of NF-κB, enhancement of microglia migration via Src/Fak/Pyk2 pathway.	[62]
SPRC	Neuroinflammation, rats, 20, 40, 80 mg/kg/day i.p., 3 days before + 9 days after LPS	Full restoration of spatial learning and memory, restoration of hippocampal H_2_S levels ↓ TNF-α, TNFR1, Aβ_1–40/42_, AβPP, ↓ IκB-α degradation, ↓ p-NF-κB p65 (effect comparable or superior to ibuprofen 40 mg/kg).	[63]
ADT-OH	Primary neural progenitor cells (NPCs) from E14.5 mouse embryonic brain (ICR), 1–20 µM	Modulation of differentiation into neurons and oligodendrocytes, suppression of astrogenesis and apoptosis, stimulation of axonal growth via ↑ β-catenin, TCF7L2, c-Myc, Ngn1/2.	[64]
ADT	Compressive SCI (T10, C57BL/6 mice), 10–40 mg/kg/day i.p. starting day 1 post-SCI, daily up to 56 days	Enhanced regeneration, ↓ scarring, neuronal death, microglial activation; ↑ vascular remodeling.	[65]
ADT-OH	BV2 microglia + LPS; primary microglia (10–50 µM); in vivo: LPS injections, mice, 50 mg/kg i.p.	↑ AMPK activation (via CaMKKβ, independent of LKB1), ↓ M1 markers (iNOS, TNF-α, IL-1β, IL-6, NO), ↑ M2 markers (Arg1, YM1, IL-10); effects abolished by CaMKKβ/AMPK inhibitor/knockout. In vivo brain: ↑ p-AMPK + M1 → M2 switch in LPS injection area.	[66]
ADT-OH	Middle cerebral artery occlusion, mice, 50 mg/kg/day 3 h after reperfusion	Maintenance of BBB permeability, ↓ lesion area, edema, Evans blue extravasation; prevention of tight junction degradation, inhibition of iNOS, IL-1β, MMP9, NOX4 via NF-κB.	[67]
ADT-OH	Ischemic stroke, mice MCAO 2 h + tPA 10 mg/kg i.v. (clinical regimen), 50 mg/kg i.p. simultaneously with tPA + daily for 7 days	↓ tPA-induced hemorrhagic transformation, ↓ BBB disruption, ↓ Akt → VEGF → MMP-9 cascade activation in endothelium, improved neurological outcomes over 7 days.	[68]
ATB-346	Controlled cortical impact models, mice, 30 µmol/kg at 1 and 6 h post-injury and once daily for 10 days	Attenuation of brain edema, neuronal death, inflammation; restoration of neurotrophic factors.	[69]
S-memantine	In vitro: SH-SY5Y, primary cortical neurons + OGD, 50 µM; in vivo: global ischemia (BCAO 40 min, mice), 25 µmol/kg	↑ intracellular H_2_S 10× higher than ACS48, ↓ toxicity (no NMDA activation or Ca^2+^ overload) ↓ infarct, ↑ survival and neurological score.	[70]
MTC	In vitro: PC12 OGD, primary neurons, 1 µM; in vivo: MCAO rats, 5 mg/kg i.p.	Activation of SOD, CAT, GPx; ↓ LDH, proapoptotic proteins, ERS, TREK-1, pro-inflammatory factors; activation of PI3K/AKT and MEK-ERK; stimulation of axonal regeneration.	[71]
NAC	Severe TBI with secondary hypoxic insult, 150 or 300 mg/kg/day daily up to 4 days	↓ acute axonal injury, early and delayed hippocampal neuronal loss (no effect on cortical lesion volume or long-term cognitive functions).	[72]
NAC + probenecid	Severe TBI in children 2–18 years (Phase I), NAC 140 → 70 mg/kg, probenecid 25 → 10 mg/kg × 4 days	Safety confirmed, stable CSF concentrations; no differences in intracranial pressure, biomarkers, or 3-month outcome.	[73]

**Table 3:** Neuroprotective effects of natural H_2_S donors in experimental models of nervous system injury. Arrows ↑ and ↓ denote increase and decrease, respectively. Abbreviations. Main active substance: H_2_S—hydrogen sulfide. Natural H_2_S donors: DADS—diallyl disulfide; DATS—diallyl trisulfide; SFN—sulforaphane; SAMe—S-adenosyl-L-methionine; GYY4137—morpholinium phosphorodithioate derivative. Main injury models: TBI—traumatic brain injury; BBB—blood–brain barrier; CCI—chronic constriction injury of the sciatic nerve; MCAo/MCAO—middle cerebral artery occlusion; cuprizone—demyelination and multiple sclerosis model. Key molecular targets and signaling pathways: Nrf2—nuclear factor erythroid 2-related factor 2; HO-1—heme oxygenase-1; AMPK—AMP-activated protein kinase; SIRT1—sirtuin 1; ULK1—UNC-51-like kinase 1; beclin1—autophagy protein Beclin-1. Inflammatory and pyroptotic pathways: NLRP3—NLRP3 inflammasome; GSDMD—gasdermin D; IL-1β—interleukin-1β; IL-17—interleukin-17; NF-κB—nuclear factor kappa B. Antioxidant systems and redox status: GSH—reduced glutathione; SOD—superoxide dismutase; TAC—total antioxidant capacity. BBB; brain edema and endothelial proteins: AQP4—aquaporin-4; vWF—von Willebrand factor; RECA-1—rat endothelial cell antigen-1. Markers of glial activation and demyelination: GFAP—glial fibrillary acidic protein. Other: DRG—dorsal root ganglia; LDH—lactate dehydrogenase.

Donor	Model, Animals, Concentration/Dose	Main Effects	Reference
DADS	Neonatal hypoxic–ischemic encephalopathy (Rice-Vannucci P7 rats: left carotid ligation + 8% O_2_ for 2.5 h) 10 mg/kg i.p. × 2 days, starting 24 h post-hypoxia	Significant ↓ infarct volume, restoration of Nissl bodies ↓ pyroptosis via NLRP3/caspase-1/GSDMD/IL-1β ↓ astrocyte activation (GFAP)	[76]
DADS + GYY4137	Neuropathic pain (CCI of sciatic nerve, mice) DADS 3.5–30 mg/kg and GYY4137 0.7–24 mg/kg i.p.	Potentiation of anti-allodynic and anti-hyperalgesic effects of μ- and δ-opioid agonists, ↑ opioid receptor expression in DRG, ↓ oxidative stress and apoptosis via H_2_S-dependent pathways	[77]
DATS + GYY4137	Oxidative cataract ex vivo (bovine lenses + 50 mM H_2_O_2_) DATS 10^−7^–10^−4^ M GYY4137 10^−7^–10^−4^ M	Prevention of lens opacification (up to 56.9% transparency recovery with DATS 10^−4^ M); full restoration of GSH and SOD activity ↓ LDH cytotoxicity by 34–36% GYY4137 more effective than DATS	[78]
DADS + SAMe	Cuprizone-induced demyelination, mice Cuprizone + DADS (100 mg/kg/day oral) and cuprizone + SAMe (20 mg/kg/day oral)	SAMe > DADS: suppression of demyelination and neuroinflammation, ↑ oligodendrocyte activity, autophagy via AMPK/SIRT1/ULK1/beclin1, ↑ GSH and TAC, ↓ fibronectin aggregation, NF-κB, IL-17	[79]
SFN	Focal ischemia (MCAo 70 min, rats) 5 mg/kg i.p. 1 h before occlusion	↑ Nrf2 and HO-1 in microvessels and perivascular astrocytes, ↓ BBB disruption, infarct volume, and neurological deficit	[80]
SFN	Severe controlled cortical TBI, rats 5 mg/kg i.p. at 1 h and 24 h post-injury	↓ brain edema and BBB permeability via Nrf2 pathway activation, improved spatial and working memory (effect only when administered within 1 h post-injury)	[81]
SFN	Severe controlled cortical TBI, rats 5 mg/kg i.p.	Blockade of AQP4 reduction in injury zone, ↑ AQP4 expression in penumbra, significant ↓ brain edema via enhanced water clearance through aquaporin channels, endothelial protection (↑ vWF, RECA-1)	[82]
SFN	Clinical trial (planned, NCT04252261)	Evaluation of 12-week SFN supplementation on cognitive functions (memory, learning) in patients with frontal lobe injury	[83]

**Table 4:** Neuroprotective effects of modern nano- and macro-carriers, hydrogels, and targeted H_2_S donors in experimental models of neurotrauma and ischemia. Arrows ↑ and ↓ denote increase and decrease, respectively. Abbreviations. Main active substance: H_2_S—hydrogen sulfide. Targeted and mitochondria-directed donors: AP39—mitochondria-targeted slow-releasing H_2_S donor; HSD-R—ROS-responsive self-degrading mitochondria-targeted H_2_S donor. Natural and hybrid donors in nanosystems: DADS—diallyl disulfide; DATS—diallyl trisulfide; SFN—sulforaphane; ADT/ADT-OH—5-(4-hydroxyphenyl)-3H-1,2-dithiole-3-thione; SHI—sulfur-containing insulin-like polypeptide. Nano- and microcarriers: MPG-ADT—peptide-targeted polymeric nanoparticles with ADT; ZnS NP—zinc sulfide nanoparticles; MION—mesoporous iron oxide nanoparticles; PEG—polyethylene glycol; LF—lactoferrin; MSN—mesoporous silica nanoparticles; MOF—metal–organic framework (Zn-CA); RBC—red blood cell membrane; GelMA—gelatin methacryloyl; PF-127—Pluronic F-127; Fe_3_S_4_—magnetic iron sulfide clusters; SF—silk fibroin; MnS@AC—manganese sulfide in activated carbon. Main injury models: TBI—traumatic brain injury; SCI—spinal cord injury; ICH—intracerebral hemorrhage; HI—hypoxic–ischemic brain injury; CPR—cardiopulmonary resuscitation; MCAO—middle cerebral artery occlusion; CCI—controlled cortical impact. Key signaling pathways and targets: mTOR—mammalian target of rapamycin; STAT3—signal transducer and activator of transcription 3; p-AMPK—phosphorylated AMP-activated protein kinase; PINK1/Parkin—mitophagy pathway; CHOP/GRP78/eIF2α—endoplasmic reticulum stress markers; ERK1/2—extracellular signal-regulated kinase 1/2; NF-κB—nuclear factor kappa B; miR-7a-5p—microRNA-7a-5p; GLT-1—glutamate transporter-1; VGLUT1—vesicular glutamate transporter 1; TrkA/TrkB—neurotrophin receptors; proNGF-p75NTR—pro-neurotrophin and its receptor; BDNF—brain-derived neurotrophic factor; bFGF—basic fibroblast growth factor. Markers of inflammation and oxidative stress: IL-6—interleukin-6; TNF-α—tumor necrosis factor alpha; MPO—myeloperoxidase; MDA—malondialdehyde; ROS—reactive oxygen species; SOD—superoxide dismutase; CAT—catalase. Markers of apoptosis and cell death: Bax—proapoptotic protein; Bcl-2—antiapoptotic protein; caspase-3—caspase-3; Bid/Apaf-1/p53—proapoptotic mediators. Cell lines and cell types: HUVEC—human umbilical vein endothelial cells; SH-SY5Y—human neuroblastoma cell line; BV2—mouse microglia; RAW264.7—mouse macrophages; RSC96—rat Schwann cells; HT22—mouse hippocampal neurons; DPSC—dental pulp stem cells; NSC—neural stem cells; H9c2—rat cardiomyocytes. Functional outcomes: MMP—mitochondrial membrane potential; CMAP—compound muscle action potential; SFI—sciatic functional index. Other: BBB—blood–brain barrier; MSC—mesenchymal stem cells; EVs—exosomes; cGAS-STING—innate immunity pathway; COX-2—cyclooxygenase-2; NSAIDs—non-steroidal anti-inflammatory drugs.

Carrier/Donor	Model, Animals, Concentration/Dose	Main Effects	Reference
G16 MPG-ADT nanoparticles (peptide-targeted polymeric nanoparticles loaded with ADT-type H_2_S donor)	Severe contusive spinal cord injury, rats, 8–56 µg/L	Prolonged H_2_S release → ↑ mTOR, STAT3 → neuronal regeneration, improved motor functions	[87]
ZnS NP (zinc sulfide nanoparticles as slow H_2_S donor)	Ischemic stroke, mice	Stable H_2_S generation → ↓ infarct, apoptosis, inflammation; ↑ neurovascularization via p-AMPK, slow H_2_S release >72 h	[88]
DATS@MION-PEG-LF (diallyl trisulfide in PEG- and lactoferrin-coated mesoporous iron oxide nanoparticles)	Cardiac arrest 5 min → CPR (SD rats), 10 mg/kg i.v. single dose immediately after successful resuscitation	↓ neuronal and cardiomyocyte apoptosis, ↓ inflammation (↓MPO), ↓ oxidative stress (↑ SOD/CAT, ↓ MDA), ↑ Bcl-2, ↓ Bax/caspase-3, full recovery of neurological function, trend toward ↑ 30-day survival	[89]
AP39 liposomes (AP39 encapsulated in liposomes, intranasal delivery)	In vitro (HUVEC + SH-SY5Y) + BBB model	Best BBB penetration among all AP39 formulations, highest cellular uptake, sustained release, complete preservation of mitochondrial function under oxidative stress	[90]
CdSe/ZnS quantum dots + Angiopep-2 peptide + CSE plasmid (combined gene/quantum system for CSE expression)	Myocardial ischemia (transferable to brain), rats/mice, 20 µL i.v. single dose just before reperfusion	Local H_2_S production → ↓ infarct, oxidative stress, mitophagy via inhibition of CHOP/GRP78/eIF2α	[91]
GelMA@LAMC hydrogel (laminin-containing gelatin methacryloyl loaded with AP39 or other H_2_S donor)	Spinal cord injury	ROS-activated H_2_S release → ↓ oxidative stress, inflammation, mitochondrial dysfunction; ↑ angiogenesis	[92]
PF-127 + OMSF@JK + stem cells (pluronic F-127 hydrogel with H_2_S donor JK-1 and MSCs)	In vitro (DPSC, RAW264.7); in vivo—rats, SCI; PF-127 17%; OMSF@JK 30/60/100 µg JK; DPSC 1 × 10^6^ cells/mL (3D gel)	High biocompatibility, stimulation of neuronal differentiation and regeneration, ↓ neuroinflammation; thermosensitive PF-127 hydrogel for local delivery of OMSF@JK and DPSC (100 µL into lesion); OMSF enhances JK loading and pH-sensitive H_2_S release	[93]
Fe_3_S_4_ ferrofluid hydrogel (magnetic Fe_3_S_4_ clusters as H_2_S source in hydrogel)	In vitro: BV2 (LPS-induced), NSC; in vivo: rats with SCI; Fe_3_S_4_ 0.01 g/mL (effective anti-inflammatory in vitro); FFH with magnetically orientable particles; NSC loaded in anisotropic FFH	↓IL-6, TNF-α, suppression of microglia/macrophage activation, inhibition of NF-κB pathway; guided axonal growth; increased axonal length; improved functional recovery after SCI	[94]
H_2_S@SF silk fibroin hydrogel (gaseous H_2_S saturated in regenerated silk fibroin)	In vitro: HT22; in vivo: severe intracerebral hemorrhage (ICH) mouse model; ~5 wt.% SF solution; local striatal injection	Prolonged H_2_S release → stabilization of water homeostasis, ↓ lesion volume and cell death in striatum, cortex, hippocampus	[95]
H_2_S@SF silk fibroin hydrogel	Mild TBI model, mice; 20 µL hydrogel applied locally to cortical surface 1 h post-CCI	↓ pyroptosis, necroptosis, edema, neuroinflammation, neurodegeneration; improved cognitive functions; much slower controlled H_2_S release than free NaHS	[96]
SF-G@Mn hydrogel (silk fibroin + glycerophosphate + Mn^2+^ as H_2_S donor)	Spinal cord injury, mice; local injection into lesion	Gradual release of H_2_S, Mn^2+^, bFGF → ↓ oxidative stress, inflammation; ↑ axonal growth and myelination; two-phase “on-demand” H_2_S release: rapid H_2_S + Mn^2+^ early + slow bFGF late	[97]
3D/GelMA/EVs (H_2_S-preconditioned mesenchymal stem cell exosomes in GelMA hydrogel)	Spinal cord injury, rats; implantation of 3D/GelMA/H_2_S-EVs scaffold directly into lesion	↑ miR-7a-5p → reduced motor deficits, sustained local release of H_2_S-EVs	[98]
mPEG-PA-PP hydrogel (temperature-sensitive polymeric hydrogel with built-in H_2_S donor)	In vivo: SD rats, complete sciatic nerve transection with 10 mm gap; in vitro: RAW264.7, RSC96 (Schwann cells), HUVEC; hydrogel injected into nerve conduit bridging 10-mm gap	ROS-sensitive H_2_S release → ↓ oxidative stress, inflammation, mitochondrial dysfunction; ↑ neuroregeneration (Schwann cells), mitochondrial function recovery (↑ complexes I/V, MMP, ATP), ↑ angiogenesis (HUVEC migration/tube formation), accelerated axonal regeneration and myelination, significant sciatic nerve function recovery (SFI, CMAP)	[99]
MnS@AC pH-sensitive hydrogel (manganese sulfide in activated carbon, H_2_S release at low pH)	Full-thickness skin wound model, mice; direct injection into wound	↓ inflammation, ↑ proliferation and angiogenesis (without cGAS-STING activation); two-level controlled release: pH-sensitive hydrogel → MnS NP release in acidic wound, then α-MnS NP → slow stable H_2_S release without burst	[100]
Zn-CA MOF (light-responsive zinc–caffeic acid metal–organic framework, photo-triggered H_2_S release)	SD rats, 10 mm sciatic nerve defect model; RSC96, RAW264.7, HUVEC	Controlled release of H_2_S and Zn^2+^ → antioxidant and anti-inflammatory effects, ↑ angiogenesis, directed cell migration, nerve and motor function recovery; photo-triggered H_2_S + Zn^2+^ release under near-infrared irradiation	[101]
DATS-MSN@PNNTBA (hypothermia-activated mesoporous silica nanoparticles with DATS and PNNTBA polymer)	In vitro: primary neonatal rat cardiomyocytes (H/R); in vivo/ex vivo: isolated C57BL/6 mouse hearts, 6 h cold storage in HT-MSN solution	Temperature-sensitive prolonged H_2_S release → cytoprotection, ↓ cardiomyocyte apoptosis, ↓ LDH,↑ BCL-2, ↓ BAX in H/R; strong protection against cold I/R injury, significant ↓ TNF-α and IL-1β	[102]
RBC-DATS-MION (DATS-loaded mesoporous iron oxide nanoparticles coated with red blood cell membrane)	In vitro: hypoxia/reoxygenation; in vivo: myocardial ischemia–reperfusion model, intravenous administration	Prolonged controlled H_2_S release into plasma and myocardium due to RBC membrane “camouflage” and extended circulation + strong antiapoptotic effects	[103]
SHI (sulfur-containing insulin-like polypeptide)	In vitro: human neuroblastoma SH-SY5Y (high glucose + 6-OHDA); in vivo: transgenic C. elegans with human α-synuclein, Drosophila melanogaster (Parkinson model)	↓ α-synuclein accumulation, ↑ dopamine transporter (DAT) expression; strong neuroprotective effects under hyperglycemia + 6-OHDA; significant motor improvement in C. elegans and Drosophila; simultaneous mitigation of neurodegenerative and hyperglycemic disorders via metabolically regulated H_2_S release at insulin action sites	[104]
AP39 (mitochondria-targeted slow H_2_S donor based on ADT)	Cerebral ischemia, rats; 90 min transient MCAO; 50 nmol/kg i.v. daily for 7 days before ischemia	↓ infarct, microglia (Iba1), pro-inflammatory cytokines, proNGF-p75NTR-caspase-3; ↑ BDNF-TrkB/NGF-TrkA; slow H_2_S release predominantly in mitochondria	[105]
AP39	90 min transient MCAO, rats; single 100 nmol/kg i.v. 10 min after reperfusion onset	↓ brain infarct volume, ↓ neurological deficit; significant ↓ extracellular glutamate in motor cortex and hippocampus, ↑ GLT-1 expression, ↓ VGLUT1; suppression of glutamate excitotoxicity; slow mitochondria-targeted H_2_S release	[106]
AP39	Photothrombotic stroke, mice; single dose 10 min post-stroke induction	↓ infarct volume, stimulation of mitophagy via PINK1/Parkin (gender-dependent effect)	[107]
AP39@Lip (AP39 encapsulated in liposomes, intranasal delivery in brain HI)	Neonatal hypoxic–ischemic brain injury (carotid ligation + hypoxia), rat pups; intranasal AP39@Lip post-HI	Mitochondrial targeting → ↓ mitochondrial dysfunction, oxidative stress, inflammation, apoptosis (blockade of ERK1/2 and caspase-1); slow mitochondria-targeted H_2_S release from liposomes with colocalization in neuronal mitochondria at 24 h	[108]
HSD-R (ROS-responsive, self-degrading, fluorescent, mitochondria-targeted H_2_S donor with triphenylphosphonium)	In vitro: H9c2 and primary neonatal rat cardiomyocytes; in vivo: rats, permanent LAD ligation; i.v. post-MI induction	Selective mitochondrial action → ↓ apoptosis (Bid/Apaf-1/p53), inflammation, ↑ angiogenesis; ROS activation → H_2_S release + red fluorescence turn-on; mitochondria-targeted, visualizable and quantifiable H_2_S release	[109]
Hybrid SFN–NSAID (sulforaphane-inspired isothiocyanates I1 and I1c—H_2_S-releasing selective COX-2 inhibitors)	LPS-induced RAW 264.7 macrophages; recombinant human COX-1/COX-2 + in silico	No cytotoxicity at 10–20 µM, very high COX-2 selectivity, slow H_2_S release; confirmed by molecular docking and 100 ns MD simulations	[110]

**Table 5:** Effects of inhibitors of endogenous H_2_S-synthesizing enzymes on the course of experimental neurotrauma and nervous system ischemia. Arrows ↑ and ↓ denote increase and decrease, respectively. Abbreviations. Main active substance: H_2_S—hydrogen sulfide. Inhibitors of H_2_S-synthesizing enzymes: AOAA—aminooxyacetic acid; PAG—propargylglycine; L-ASP—L-asparagine; benserazide—selective CBS inhibitor; NSC4056—aurintricarboxylic acid; oximic hydrazide 1—first highly selective membrane-permeable CSE inhibitor. H_2_S-synthesizing enzymes: CBS—cystathionine-β-synthase; CSE—cystathionine-γ-lyase; 3-MST—3-mercaptopyruvate sulfurtransferase. Other enzymes and cofactors: PLP—pyridoxal-5′-phosphate; MGL—methionine-γ-lyase; ALT—alanine aminotransferase; SIRT1—sirtuin 1. Injury models and conditions: TBI—traumatic brain injury; HI—hypoxic–ischemic brain injury; I/R—ischemia/reperfusion; MCAO—middle cerebral artery occlusion; OGD/R—oxygen-glucose deprivation/reoxygenation; SD—spreading depolarization; GBM—glioblastoma; IDHwt—wild-type isocitrate dehydrogenase; IDH1mut—mutant isocitrate dehydrogenase 1. Key signaling pathways and molecules: Nrf2—nuclear factor erythroid 2-related factor 2; p-STAT3—phosphorylated STAT3; NAD^+^/NADH—oxidized/reduced nicotinamide adenine dinucleotide; RhoA/ROCK—RhoA-associated kinase; eNOS—endothelial NO synthase; NO—nitric oxide; sGC—soluble guanylate cyclase; HIF-1α—hypoxia-inducible factor 1α; VEGF—vascular endothelial growth factor. Inflammatory markers: iNOS—inducible nitric oxide synthase; COX-2—cyclooxygenase-2; TNF-α—tumor necrosis factor alpha; IL-1β—interleukin-1β; IL-6—interleukin-6; CXCL11—C-X-C motif chemokine ligand 11. Antioxidant defense and oxidative stress: GSH—reduced glutathione; ROS—reactive oxygen species. Damage marker proteins: p53—tumor suppressor p53; APP—amyloid precursor protein. Cell lines: BV2—mouse microglial cell line; HEK293T—human embryonic kidney cells; COS-7—African green monkey kidney cells; RAW264.7—mouse macrophages; KYSE450—human esophageal cancer; A549—human lung adenocarcinoma; HCT8—human colorectal cancer; NCH644; NCH421k; NCH1681; NCH551b; NCH612—patient-derived glioma lines. Other: LPS—lipopolysaccharide; miR-9-5p—microRNA-9-5p; MAP—mean arterial pressure; K_ATP channels—ATP-sensitive potassium channels; BK channels—large-conductance calcium-activated potassium channels.

Inhibitor	Model, Animals, Concentration/Dose	Main Observed Effects	Reference
AOAA	In vivo: mice, systemic LPS 250 µg/kg/day for 7 days; in vitro: BV2 microglia	↓ neuroinflammation, ↓ microglia activation, iNOS, COX-2, IL-1β, IL-6, TNF-α via altered NAD^+^/NADH ratio and ↓ p-STAT3	[113]
AOAA	In vivo: neonatal HI (right carotid ligation + 8% O_2_ 1 h), mice, 5 mg/kg i.p. at 24, 48, 72 h post-HI; in vitro: BV2 + LPS (1 µg/mL)	Blocks L-cysteine-dependent neuroprotection, ↓ miR-9-5p and CBS, ↑ TNF-α, IL-1β, CXCL11	[114]
AOAA	Cerebral ischemia/reperfusion, primary astrocytes, OGD/R model, mice, transient MCAO	Blocks neuroprotective effects of TFR (inhibits astrogliosis and RhoA/ROCK pathway)	[115]
AOAA	Isolated bovine neural retina; model: 100 µM H_2_O_2_ for 10 min	No effect on cannabinoid-mediated neuroprotection (unlike 3-MST inhibitor)	[116]
AOAA	Spreading depolarization (SD), rats	Complete suppression of brain H_2_S fluctuations	[117]
AOAA	Focal ischemic stroke (MCAO 90–120 min), rats	↓ hypothalamic H_2_S → ↑ arterial pressure and sympathetic activity	[38]
AOAA	TBI (controlled cortical impact), mice; axotomy in crayfish mechanoreceptor neuron	Enhances neuronal and glial death, ↑ p53, iNOS, APP	[20,21]
Octreotide + PAG	Controlled cortical TBI (prefrontal cortex), rats, i.p.	Blocks octreotide neuroprotection, ↓ H_2_S, Nrf2, ↑ TNF-α	[118]
PAG + AOAA	Global cerebral I/R (bilateral carotid occlusion 20 min) + vascular dementia, mice	Blocks protective effects, ↓ H_2_S, CBS, CSE, Nrf2, ↑ oxidative damage	[119]
PAG	Newborn piglets, cranial window, pial arterioles	↓ endothelial H_2_S generation, blocks vasodilation via K_ATP and BK channels	[120]
PAG	Patient-derived glioma spheres (IDHwt GBM and IDH1mut astrocytoma/oligodendroglioma); orthotopic xenografts in NSG mice (NCH1681)	IDH1-mutant astrocytomas critically depend on CSE/H_2_S for GSH synthesis and antioxidant defense despite impaired NADPH/NADP^+^ PAG or siRNA → drastic ↓ GSH, ↑ ROS, ↑ cell death only in IDH1mut, not IDHwt; in vivo: PAG significantly slows orthotopic IDH1mut glioma growth	[121]
PAG	Ex vivo: isolated penetrating arterioles from aged mice (9–12 mo) and human brain biopsies	CSE/H_2_S unexpectedly promotes high-K^+^-induced vasoconstriction; PAG markedly attenuates K^+^-induced constriction (fully reversed by NaHS) Mechanism: H_2_S → eNOS activation → NO → sGC → enhanced constriction	[122]
Oximic hydrazide 1 (novel highly selective CSE inhibitor)	Recombinant rCSE, hCSE in HEK293T lysates, live COS-7 cells transfected with hCSE	First highly selective membrane-permeable CSE inhibitor; suitable for precise pharmacological separation of CSE vs. CBS/3-MST functions; completely suppresses H_2_S production in live cells, covalently binds PLP (confirmed by crystallography); high selectivity: no inhibition of CBS, 3-MST, MGL, ALT PAG far less selective	[123]
L-ASP + PAG	Primary rat cerebrovascular endothelial cells; isolated rat basilar artery (myography)	↓ acetylcholine-induced H_2_S generation and vasodilation, activation of RhoA-ROCK	[124]
Benserazide (selective CBS inhibitor)	KYSE450 (esophageal), A549 (lung), HCT8 (colorectal) cells; axillary lymph node xenografts in mice	↓ SIRT1 sulfhydration, ↓ HIF-1α/VEGF, ↓ H_2_S by ~90%, binds CBS active site	[125]
NSC4056 (aurintricarboxylic acid)—highly potent reversible CSE inhibitor	Hemorrhagic shock rats, 50 mg/kg i.v. (single); recombinant hCSE, HEK293T and RAW264.7 cells	Most potent and selective CSE inhibitor: ↓ endogenous H_2_S in macrophages and HEK293T (WT CSE), no effect on mutants or CBS; in vivo: completely prevents hypotension in hemorrhagic shock (MAP ↑ to 65 mmHg vs. 35 in control), ↓ plasma H_2_S, ↑ homocysteine	[126]

**Table 6:** Comparative characteristics of H_2_S donors for neuroprotection in preclinical models of CNS and PNS injury: from classical inorganic salts to modern targeted and stimulus-responsive delivery systems. Arrows ↑ and ↓ denote increase and decrease, respectively. Abbreviations. Main active substance: H_2_S—hydrogen sulfide. Inorganic donors: NaHS—sodium hydrosulfide; Na_2_S—sodium sulfide; Na_2_S_3_—sodium trisulfide; STS—sodium thiosulfate. Thiol-activated fluorescent donors: COS/H_2_S—thiol-activated H_2_S donor based on carbonyl sulfide; AlaCOS—alanine-containing thiol-activated H_2_S donor with coumarin fluorophore. Classical slow synthetic donors: GYY4137—4-methoxyphenyl(morpholino)phosphinodithioate; SPRC/ZYZ-802—S-propargyl-L-cysteine. Hybrid donors based on approved drugs: ACS67—latanoprost-H_2_S hybrid; ACS84—L-DOPA-H_2_S hybrid; ATB-346—naproxen-H_2_S hybrid; S-memantine—memantine-H_2_S hybrid. Dithiolethione donors (ADT family): ADT-OH/ADT—5-(4-hydroxyphenyl)-3H-1,2-dithiole-3-thione; MTC—S-allyl-L-cysteine-gallic acid conjugate. Natural donors: DADS—diallyl disulfide; DATS—diallyl trisulfide; SAMe—S-adenosyl-L-methionine; SFN—sulforaphane. Mitochondria-targeted donors: AP39—mitochondria-targeted slow H_2_S donor with triphenylphosphonium cation; AP39@Lip—AP39 in liposomes; HSD-R—ROS-responsive self-degrading mitochondria-targeted H_2_S donor. Nanocarriers and stimulus-responsive systems: ZnS NP—zinc sulfide nanoparticles; DATS@MION-PEG-LF—diallyl trisulfide in PEG- and lactoferrin-coated mesoporous iron oxide nanoparticles; RBC-DATS-MION—diallyl trisulfide in red blood cell membrane-coated mesoporous iron oxide nanoparticles; Zn-CA MOF—light-responsive zinc-caffeic acid metal–organic framework; MnS@AC—pH-responsive manganese sulfide in activated carbon; DATS-MSN@PNNTBA—hypothermia-activated mesoporous silica nanoparticles with diallyl trisulfide. Hydrogels and 3D scaffolds: GelMA@LAMC—laminin-containing gelatin methacryloyl hydrogel with AP39; PF-127—Pluronic F-127; OMSF@JK—silica nanoparticles with JK-1 donor; Fe_3_S_4_ FFH—magnetic ferrofluid hydrogel with iron sulfide clusters; H_2_S@SF—gaseous H_2_S-saturated silk fibroin hydrogel; SF-G@Mn—silk fibroin hydrogel with glycerophosphate and manganese ions; 3D/GelMA/H_2_S-EVs—three-dimensional gelatin methacryloyl hydrogel with H_2_S-preconditioned mesenchymal stem cell exosomes; mPEG-PA-PP—ROS-responsive polymeric hydrogel. Main injury models: TBI—traumatic brain injury; SCI—spinal cord injury; ICH—intracerebral hemorrhage; BBB—blood–brain barrier. Key molecular targets and signaling pathways: K_ATP channels—mitochondrial ATP-sensitive potassium channels; ROCK2—Rho-associated kinase 2; NR1/NMDAR—subunit 1 of N-methyl-D-aspartate receptor; p53—tumor suppressor p53; NLRP3—NLRP3 inflammasome; caspase-3—caspase-3; MMP-9—matrix metalloproteinase-9; occludin—tight junction protein; EAAT2/GLT-1—excitatory amino acid transporter 2/glutamate transporter-1; VGLUT1—vesicular glutamate transporter 1; BDNF—brain-derived neurotrophic factor; TrkB/TrkA—tyrosine kinase receptors B and A; ARC—activity-regulated cytoskeleton-associated protein; PSD-95—postsynaptic density protein 95; NF-κB—nuclear factor kappa B; p38 MAPK—p38 mitogen-activated protein kinase; Nrf2—nuclear factor erythroid 2-related factor 2; ARE—antioxidant response element; HO-1—heme oxygenase-1; AQP4—aquaporin-4; AMPK—AMP-activated protein kinase; CaMKKβ—calcium/calmodulin-dependent protein kinase kinase β; SIRT1—sirtuin 1; ULK1—UNC-51-like kinase 1; beclin-1—Beclin-1; PINK1/Parkin—mitophagy pathway; MMP—mitochondrial membrane potential; ROS—reactive oxygen species; GSH—reduced glutathione; SOD—superoxide dismutase; VEGF—vascular endothelial growth factor; bFGF—basic fibroblast growth factor; miR-7a-5p—microRNA-7a-5p; cGAS-STING—cyclic GMP-AMP synthase—stimulator of interferon genes pathway. Markers of cell death: TUNEL—terminal deoxynucleotidyl transferase dUTP nick end labeling; Bax/Bad—proapoptotic Bcl-2 family proteins; MLKL/RIP1/RIP3—necroptosis markers; GSDMD—gasdermin D. Tests of cognitive and motor function: MWM—Morris water maze; NOR—novel object recognition; ERG—electroretinography.

Class and Subclass of Donors	Representative Compounds	H_2_S Release Kinetics	Primary Molecular Targets and Key Mechanisms	Main Neuroprotective Effects in Models of TBI, Ischemia, Neuropathy, Demyelination, SCI, ICH	Advantages	Limitations/Translational Challenges	References
I. Inorganic donors							
I.A Fast sulfide salts	NaHS, Na_2_S, Na_2_S_3_	Seconds–minutes (“burst”)	Mitochondrial K_ATP channels, restoration of NR1/NMDAR disulfide bonds, ↓ ROCK2 (Thr436/Ser575),↓p53 (van der Waals Arg248, pH-dependent), caspase-3 persulfidation (Cys163),↓ MMP-9, occludin,↑ EAAT2	↓ brain edema, BBB permeability, infarct volume, apoptosis (TUNEL), oxidative stress; ↑ BDNF, ARC, PSD-95, synaptic plasticity; restoration of cognitive/motor functions; protection of dopaminergic neurons from necroptosis (↓ IL-17/MLKL)	Fastest effect in acute phase (min–h); cheap and widely available	Very narrow therapeutic window, cytotoxicity risk > 200–500 µM, H_2_S “spike”	[20,21,26,27,28,29,30,31,32,33,34,35,36,37,38,39,40,41,42]
I.B Sodium thiosulfate (STS)	Sodium thiosulfate	Hours, mediated by oxidation to thiosulfate → persulfidation	Caspase-3 persulfidation (Cys163), ↓ p53 (pH-dependent), ↑ GSH in glia, ↓ NF-κB/p38, VEGF-dependent peripheral angiogenesis	↓ neuronal/astrocyte apoptosis, BBB stabilization after cardiac arrest, protection from OGD/R in vitro/in vivo	Safer profile, pH-sensitive “metabolic sensor”, fully reproduces Na_2_S effects	Poor penetration through intact BBB, effect only in periphery or when BBB disrupted	[21,42,43,44]
I.C Thiol-activated fluorescent donors	COS/H_2_S, AlaCOS	Thiol-dependent, with fluorescence turn-on	Direct H_2_S release in presence of Cys/GSH, APN activation (AlaCOS)	Tissue-specific delivery, H_2_S visualization in macrophages and wounds	First real-time H_2_S tracking system in vivo	Proof of concept only, no CNS data yet	[45,47]
II. Synthetic organic slow donors							
II.A Classical slow donors	GYY4137, SPRC (ZYZ-802)	Hours–days, thiol-independent (GYY4137) or CBS-dependent (SPRC)	↓ NLRP3 inflammasome, ↓ Bax/Bad/caspase-3/MLKL/RIP1/3, ↓ NF-κB/MAPK, ↑ CD24/Src/Fak/Pyk2, ↓ autophagic occludin degradation	↓ infarct, edema, Aβ deposition, neuroinflammation; improved memory (MWM, NOR), locomotion; BBB protection	Prolonged protection without toxicity peak	Dose-dependent efficacy (SPRC)	[50,51,52,53,54,55,60,61,62,63]
II.B Drug-hybrid donors	ACS67 (latanoprost-H_2_S), ACS84 (L-DOPA-H_2_S), ATB-346 (naproxen-H_2_S), S-memantine	Hours–days, thiol-activated	↑ Nrf2 → GSH/SOD, NMDA blockade (S-memantine), COX inhibition + H_2_S	Retinal protection, dopaminergic neuron protection (MPTP, 6-OHDA), ↓ TBI edema, improved ERG	Known PK profile of parent drug	Complex synthesis, preclinical only	[56,57,58,59,69,70]
II.C Dithiolethione (ADT family)	ADT-OH, ADT, MTC	Slow, thiol-dependent	↑ AMPK/CaMKKβ → M1 → M2 microglia switch, ↑ β-catenin/TCF7L2/c-Myc/Ngn1/2, ↑ PI3K/AKT, MEK-ERK	Microglia M2 polarization, axonal regeneration, ↓ SCI scarring, BBB protection during tPA-induced hemorrhage	High regenerative activity	Scale-up complexity	[64,65,66,67,68,71]
III. Natural donors							
III.A Garlic polysulfides	DADS, DATS	Slow, strictly thiol-dependent; DATS ≫ DADS	↓ NLRP3/caspase-1/GSDMD (pyroptosis), ↑ μ/δ-opioid receptors in DRG	↓ pyroptosis, demyelination, neuropathic pain; potentiation of opioid analgesia	Food-grade safety, opioid synergy	Low stability, variable content	[76,77,78]
III.B SAMe	S-adenosyl-L-methionine	Indirect (transsulfuration)	↑ AMPK/SIRT1/ULK1/beclin-1 (autophagy)	Suppression of cuprizone-induced demyelination, ↑ GSH/TAC	Physiological metabolite	Slow action	[79]
III.C Isothiocyanates (sulforaphane)	SFN	Very slow (days) + strong Nrf2 activation	Nrf2-ARE → ↑ HO-1/AQP4, BBB protection	↓ TBI edema and BBB permeability, memory improvement; ongoing clinical trial (NCT04252261)	High safety, long-term oral use	Low bioavailability	[80,81,82,83]
IV. Innovative hybrid and targeted systems							
IV.A Mitochondria-targeted	AP39, AP39@Lip (intranasal), HSD-R	Slow, >90% mitochondrial, ROS-responsive (HSD-R)	↑ PINK1/Parkin (mitophagy), ↑ complexes I/V, MMP, ↑ BDNF-TrkB/NGF-TrkA, ↑ GLT-1/↓ VGLUT1, ↓ ERK1/2, caspase-1	↓ infarct, excitotoxicity, oxidative stress; BBB crossing (intranasal); gender-dependent mitophagy	Highest efficacy at nmol/kg level	Preclinical only	[90,105,106,107,108,109]
IV.B Nanocarriers and stimulus-responsive systems	ZnS NP, DATS@MION-PEG-LF, RBC-DATS-MION, Zn-CA MOF (light), MnS@AC (pH), DATS-MSN@PNNTBA (temperature)	Prolonged + “on-demand” (ROS/pH/light/magnetic/temperature), >72 h–weeks	↓ NF-κB, cGAS-STING, ↑ p-AMPK, ↑ angiogenesis	↓ infarct, apoptosis, inflammation; full neurological recovery after cardiac arrest; release visualization	Personalized delivery, BBB penetration	High cost, unknown long-term safety	[88,89,100,101,102,103]
IV.C Hydrogels and 3D scaffolds	GelMA@LAMC, PF-127+OMSF@JK+MSC, Fe_3_S_4_ FFH, H_2_S@SF, SF-G@Mn, 3D/GelMA/H_2_S-EVs, mPEG-PA-PP	Long-term (weeks), local, ROS/pH/magnetic-sensitive	↑ miR-7a-5p, BDNF/bFGF, guided axonal growth, M2 microglia switch	Best regeneration in SCI and ICH, myelination, angiogenesis, water homeostasis stabilization	Synergy with stem cells/exosomes, minimal systemic toxicity	Complex manufacturing, implantation required	[92,93,94,95,96,97,98,99]

## Data Availability

No new data were created or analyzed in this study. Data sharing is not applicable to this article.

## Supplementary Materials

The following supporting information can be downloaded at https://www.mdpi.com/article/10.3390/ijms262411842/s1.
