# Supplementary material for: Inorganic, Synthetic, Natural, and Innovative Hybrid Hydrogen Sulfide Donors and Inhibitors of Its Biosynthesis in the Treatment of Central and Peripheral Nervous System Injuries: A Systematic Analytical Review
Source: Int J Mol Sci. 2025 Dec 8;26(24):11842. doi: 10.3390/ijms262411842 (PMC12732372; doi:10.3390/ijms262411842)
Supplement: Supplementary file 1 [file ijms-26-11842-s001.zip › ijms-3991254-supplementary.pdf]

Table S1. Expanded Search Strategy for Each Database. Note: * — wildcard search for word root; "" — exact keyword; () — keyword grouping; "AND" — both terms required; "OR" — either term required.

Database	Search Strategy
PubMed	((("hydrogen sulfide" OR "H2S" OR "sulphide" OR "sulfide" OR "sulfane sulfur" OR "sulfhydration" OR "persulfidation") OR ("H_2S donor" OR "NaHS" OR "sodium hydrosulfide" OR "GYY4137" OR "AP39" OR "AP123" OR "ZYZ-802" OR "SPRC" OR "ADT-OH" OR "diallyl trisulfide" OR "DATS" OR "thiosulfate" OR "sodium thiosulfate" OR "COS donor")) AND ("traumatic brain injury" OR "TBI" OR "brain trauma" OR "cerebral trauma" OR "head injury" OR "spinal cord injury" OR "SCI" OR "neurotrauma" OR "peripheral nerve injury" OR "axotomy" OR "neuropathic pain" OR ""neuroprotection" OR "neuroprotective agent" OR "neuronal survival" OR "glial activation" OR "blood-brain barrier" OR "BBB dysfunction" OR "oxidative stress" OR "inflammation" OR "apoptosis" OR "necroptosis" OR "pyroptosis" OR "ferroptosis" OR "autophagy" OR "mitochondrial dysfunction" OR "endoplasmic reticulum stress" OR "synaptic plasticity" OR "cognitive impairment" OR ""motor deficit" OR "dendritic arborization" OR "microglial polarization" OR "astrocyte reactivity"))
Scopus	TITLE-ABS-KEY(("hydrogen sulfide" OR "H_2S" OR "sulphide" OR "sulfide" OR "sulfane sulfur" OR "sulfhydration" OR "persulfidation") OR ("H2S donor" OR "NaHS" OR "sodium hydrosulfide" OR "GYY4137" OR "AP39" OR "AP123" OR "ZYZ-802" OR "SPRC" OR "ADT-OH" OR "diallyl trisulfide" OR "DATS" OR "thiosulfate" OR "sodium thiosulfate" OR "COS donor")) AND TITLE-ABS-KEY(("traumatic brain injury" OR "TBI" OR "brain trauma" OR "cerebral trauma" OR "head injury" OR "spinal cord injury" OR "SCI" OR "neurotrauma" OR "peripheral nerve injury" OR "axotomy" OR "neuropathic pain" OR "neuroprotection" OR "neuroprotective agent" OR "neuronal survival" OR "glial activation" OR "blood-brain barrier" OR "BBB dysfunction" OR "oxidative stress" OR "inflammation" OR "apoptosis" OR "necroptosis"" OR "pyroptosis" OR "ferroptosis" OR "autophagy" OR "mitochondrial dysfunction" OR "endoplasmic reticulum stress" OR "synaptic plasticity" OR "cognitive impairment" OR "motor deficit" OR "dendritic arborization" OR "microglial polarization" OR "astrocyte reactivity"))
Web of Science	TOPIC: (*hydrogen sulfide* OR *H_2S* OR *sulphide* OR *sulfide* OR *sulfane sulfur* OR *sulfhydration* OR *persulfidation*) OR TOPIC: (*H_2S donor* OR *NaHS* OR *sodium hydrosulfide* OR *GYY4137* OR *AP39* OR *AP123* OR *ZYZ-802* OR *SPRC* OR *ADT-OH* OR *diallyl trisulfide* OR *DATS* OR *thiosulfate* OR *sodium thiosulfate* OR *COS donor*) AND TOPIC: (*traumatic brain injury* OR *TBI* OR *brain trauma* OR *cerebral trauma* OR *head injury* OR *spinal cord injury* OR *SCI* OR *neurotrauma* OR *peripheral nerve injury* OR *axotomy* OR *neuropathic pain* OR *neuroprotection* OR *neuroprotective agent* OR *neuronal survival* OR *glial activation* OR *blood-brain barrier* OR *BBB dysfunction* OR *oxidative stress* OR *inflammation* OR *apoptosis* OR *necroptosis* OR *pyroptosis* OR *ferroptosis* OR *autophagy* OR *mitochondrial dysfunction* OR *endoplasmic reticulum stress* OR *synaptic plasticity* OR *cognitive impairment* OR *motor deficit* OR *dendritic arborization* OR *microglial polarization* OR *astrocyte reactivity*)

Table S2. Characteristics and distribution of included studies by thematic categories. Values in square brackets indicate the primary references assigned to each thematic category, while values in parentheses denote additional references that recur across multiple sections. The total number of unique studies included across all categories is n = 186.

Section	Category	Number	List
Introduction	Articles	19 (3)	[1-18],[22] (19-21)
Materials and Methods	Articles	1	[23]
Inorganic H_2S Donors	Experimental	24	[19-21],[26-45],[47]
	Reviews	3	[24,25],[46]
Organic H_2S Donors	Experimental	24	[50-73]
	Reviews	2 (2)	[48,49] (24,25)
Natural H_2S Donors	Experimental	8	[76-83]
	Reviews	2	[74,75]
Innovative Multicomponent Hybrid H_2S Donors	Experimental	24	[87-110]
	Reviews	3	[84-86]
Inhibitors of H_2S-Synthesizing Enzymes	Experimental	14 (3)	(20,21,38),[113-126]
	Reviews	2	[111,112]
Discussion	Articles	66 (76)	[127-186] (7, 8, 10, 12, 13, 17, 18, 19, 20, 21, 26, 27, 29, 30, 31, 32, (33–36), 38, 40, 42, (44–47), 50, 51, 53, (56–63), (64–74), (76–83), (87–95), (97–108), 114, 115, 117, 118, 119, (121–126))
	Reviews	32	[128-131], [133], [137], [138], [141-143], [149], [152], [154-156], [161], [163], [164], [167], [172], [173], [175-177], [178], [180], [182-184], [186, 187], [190]
	Experimental	34	[127], [132], [134-136], [139], [140], [144-148], [150-153], [157-160], [162], [165], [166], [168], [169], [170], [171], [174], [179], [181], [185], [188-189], [191-192]
